# Iron and Copper Alter the Oxidative Potential of Secondary Organic Aerosol: Insights from Online Measurements and Model Development

**DOI:** 10.1021/acs.est.3c01975

**Published:** 2023-08-25

**Authors:** Steven J. Campbell, Battist Utinger, Alexandre Barth, Suzanne E. Paulson, Markus Kalberer

**Affiliations:** § Department of Environmental Sciences, University of Basel, Klingelbergstrasse 27, 4057 Basel, Switzerland; ‡ Department of Atmospheric and Oceanic Sciences, University of California at Los Angeles, 520 Portola Plaza, Los Angeles, California 90095, United States

**Keywords:** aerosol particles, oxidative potential, secondary organic aerosol, reactive oxygen species, ascorbic acid, DCFH, hydroxyl radicals

## Abstract

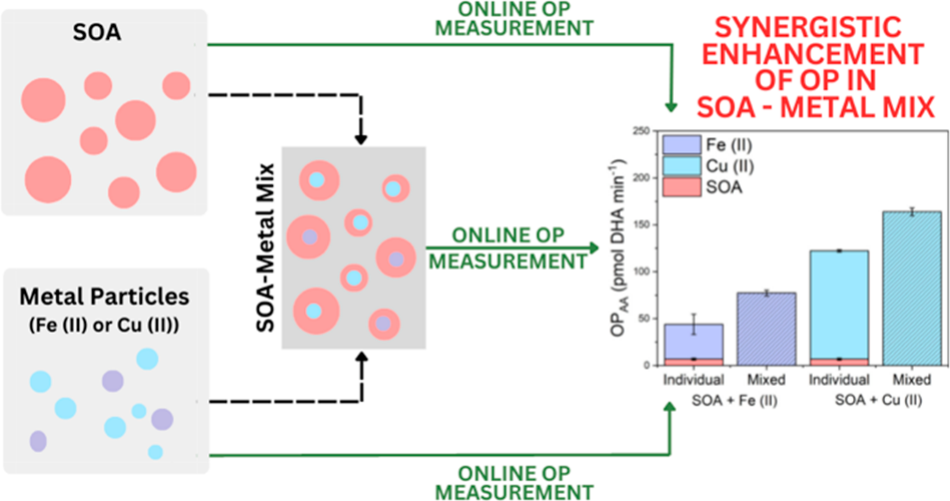

The oxidative potential (OP) of particulate matter has been widely suggested as a key metric for describing atmospheric particle toxicity. Secondary organic aerosol (SOA) and redox-active transition metals, such as iron and copper, are key drivers of particle OP. However, their relative contributions to OP, as well as the influence of metal–organic interactions and particulate chemistry on OP, remains uncertain. In this work, we simultaneously deploy two novel online instruments for the first time, providing robust quantification of particle OP. We utilize online AA (OP_AA_) and 2,7-dichlorofluoroscein (ROS_DCFH_) methods to investigate the influence of Fe(II) and Cu(II) on the OP of secondary organic aerosol (SOA). In addition, we quantify the OH production (OP_OH_) from these particle mixtures. We observe a range of synergistic and antagonistic interactions when Fe(II) and Cu(II) are mixed with representative biogenic (β-pinene) and anthropogenic (naphthalene) SOA. A newly developed kinetic model revealed key reactions among SOA components, transition metals, and ascorbate, influencing OP_AA_. Model predictions agree well with OP_AA_ measurements, highlighting metal–ascorbate and −naphthoquinone–ascorbate reactions as important drivers of OP_AA_. The simultaneous application of multiple OP assays and a kinetic model provides new insights into the influence of metal and SOA interactions on particle OP.

## Abstract 2

We quantify the oxidative potential of biogenic and anthropogenic secondary organic aerosol and show synergistic enhancement of their oxidative potential when mixed with Fe(II) and Cu(II) particles.

## 1 Introduction

Decades of large-scale epidemiological studies have consistently linked exposure to airborne particulate matter with an aerodynamic diameter <2.5 μm (PM_2.5_) with adverse health outcomes.^1,2^ The World Health Organization recently updated guideline annual exposure limits for PM_2.5_ from 10 to 5 μg m^–3^. With this recent update, 99% of the world’s population now lives in places that exceed these guideline limits. However, the specific properties of particles which are most damaging to human health, such as their size, shape and chemical composition, and their mechanisms of toxicity upon exposure, remain largely uncertain.^3^

The promotion of oxidative stress, defined as an imbalance of the oxidant to antioxidant ratio in favor of the former, overwhelming the lung’s natural antioxidant defenses upon particle deposition, has been widely suggested as a key mechanism describing particle toxicity. Reactive oxygen species (ROS), a term typically referring to the hydroxyl radical (OH), hydroperoxyl radical (HO_2_), superoxide (O_2_^•–^), hydrogen peroxide (H_2_O_2_), and in some cases organic peroxides (ROOH) and organic radicals, are key drivers of oxidative stress.^4^ The catalytic production of ROS by redox-active particle components with subsequent depletion of antioxidants is defined as oxidative potential (OP).^3^

There are a range of acellular chemical assays that are utilized to measure particle OP and particle-bound ROS, including but not limited to: 2,7-dichlorofluoroscein (DCFH); the ascorbic acid (AA) assay; the terephthalate assay (TA); and the dithiothreitol (DTT) assay. These assays are sensitive to a broad range of chemical components that likely contribute to particle OP, including H_2_O_2_ and organic peroxides (DCFH),^5,6^ redox-active transition metals (DTT/AA),^7−10^ quinones (DTT/AA), and hydroxyl radicals (TA).^11−14^ Several studies in the literature have demonstrated that total organic carbon (OC),^15,16^ as well as specific organic fractions including water-soluble organic carbon (WSOC) and secondary organic aerosol (SOA),^17,18^ quinones,^11,12^ and humic-like substances (HULIS),^19^ as well as redox-active transition metals including Cu, Fe, and Mn,^7−10^ are key drivers of particle OP. However, only a few studies have probed the chemical interaction of these species.^20−24^ Processes such as metal–organic ligand formation, influencing metal solubility and redox chemistry,^23,25^ and chemical reactions between organic aerosol components with metals, such as Fenton-like peroxide decomposition by Fe(II),^26^ likely change the oxidative properties of these key species. Thus, metal–organic chemistry in particles likely influences the physical and chemical properties of PM, including OP, and subsequently the health implications of these particle components.

Traditional methods for measuring PM OP have largely relied on the collection of particle samples on filters, with analysis occurring typically several hours, days, weeks, or even months after particle collection. Offline sampling may then underestimate OP, as highly reactive components such as organic peroxides can decompose prior to analysis.^5^ In a recent study by Zhang et al.,^27^ we showed that up to 90% of particle-bound ROS are lost prior to offline analysis due to the ∼24 h time delay between particle collection on a filter prior to analysis. This emphasizes the importance of online direct-to-reagent methods for robust quantification of particle OP, in particular for SOA, which can be rich in organic peroxides that have a range of lifetimes from approximately minutes to several days, depending on the peroxide molecular structure and multiphase loss processes at play.^28^

Recently, we developed an online methodology that can directly measure particle OP with immediate liquid extraction in the presence of the OP assay, with a time resolution of approximately 10 min. We have developed two iterations of this instrument: the Online Particle-bound ROS Instrument (OPROSI),^6^ which utilizes the DCFH assay, and the Online Oxidative Potential Ascorbic Acid Instrument (OOPAAI),^29,30^ another instrument version which adopts an ascorbic acid based assay. These instruments allow highly time-resolved, accurate quantification of ROS_DCFH_ (OPROSI) and OP_AA_ (OOPAAI), also capturing short-lived ROS and OP-active components, which filter-based methods may underestimate. Thus, the simultaneous application of two unique online methods provides robust quantification of particle oxidative properties which contribute to particle OP.

In this work, we deploy the OPROSI and OOPAAI simultaneously for the first time, probing both online ROS_DCFH_ and OP_AA_. We investigate the effects of mixing redox-active transition metals (Fe(II) and Cu(II), amongst some of the most abundant metals in ambient aerosol particles) with biogenic (BSOA, using β-pinene as the precursor) and anthropogenic (NSOA, using naphthalene as the precursor) SOA particles. BSOA and NSOA have significantly different chemical composition, and originate from different sources in the atmosphere. In addition, OH measurements (OP_OH_) were performed on filters collected simultaneously with online measurements. The metals produce a range of synergistic and antagonistic effects on ROS_DCFH,_, OP_AA_, and OP_OH_. We also develop a detailed kinetic model, building on our previous work by Shen et al.,^31^ incorporating chemistry describing the reaction of naphthoquinones with ascorbic acid, ROS, Fe(II), and Cu(II), as well as organic peroxide chemistry.

## 2 Materials and Methods

### 2.1 Particle production and Online Measurement of Aerosol Particle ROS_DCFH_ and OP_AA_

Aerosol particles in this study were produced using a nebulizer for Fe(II) and Cu(II) seed particles and an organic coating unit (OCU)^32^ for BSOA and NSOA, which is described in detail in Sections S1.2 and S1.5 in the Supporting Information (see Figure S1 for a schematic of the experimental setup).

Particle masses were broadly in the range of 245–408 μg m^–3^ for SOA and 5–35 μg m^–3^ Fe(II) and Cu(II) nebulized aerosol particles (Table S1, Section S1.5). Experiments where SOA and metals were mixed were in the same mass range, with a ∼10:1 ratio for SOA:Fe(II) and a ∼50:1 ratio for SOA:Cu(II), aiming to broadly represent metal–SOA ratios observed in previous studies in polluted urban environments, where SOA is generally a far greater contributor to particle mass than Fe(II) and Cu(II).^9,33^ For experiments involving mixtures of both SOA and metal particles, the particles are well mixed as opposed to two particle populations in parallel, as evidenced by the one mode observed in the particle size distribution in Figure S2.

Online measurements of aerosol particle OP were performed by using two instruments developed within our group: the online particle-bound ROS instrument (OPROSI, ROS_DCFH_), based on the chemistry of DCFH, and the Online Oxidative Potential Ascorbic Acid Instrument (OOPAAI, OP_AA_), which is a modified version that utilizes a fluorescence-based AA assay. Detailed descriptions of the instruments can be found in Wragg et al.,^6^ Campbell et al.,^29^ and Utinger et al.^30^ Additional information is also given in Section S1.3 and S1.4 in the Supporting Information, respectively, and a brief operational overview will be provided here.

Briefly, the OPROSI is operated by continuously drawing the aerosol sample into the instrument at a flow rate of 7 L min^–1^ through an activated charcoal denuder to remove gas-phase artifacts such as VOCs, O_3_ ,and H_2_O_2_,^34^ before entering a home-built particle sampler. Particles are collected onto a wetted filter continuously sprayed with a solution of horseradish peroxidase (HRP) in 10% PBS buffer. This immediately reacts with ROS present in the particles, such as ROOH and ROOR, or H_2_O_2_ produced by SOA chemistry and is collected in a 10 mL liquid reservoir. The HRP solution is then immediately mixed with 2,7-dichlorofluoroscein (DCFH), which is subsequently oxidized to form a fluorescent product DCF by the ROS-HRP solution in a reaction bath maintained at 37 °C for 15 min. DCF is then quantified via fluorescence spectroscopy (λ_ex_ = 470 nm, λ_em_ = 522 nm). The fluorescence response of the instrument is calibrated with known concentrations of hydrogen peroxide (H_2_O_2_), and thus, ROS_DCFH_ concentrations are expressed in H_2_O_2_ equivalent concentrations per unit volume (m^–3^) or per unit particle mass (μg^–1^). The DCFH assay has demonstrated sensitivity in particular to H_2_O_2_, organic peroxides and organic hydroperoxides.^5,6^ The direct-to-liquid sampling and high time resolution of this instrument captures short-lived ROS (typically peroxide) components, which react within seconds after sampling with HRP.^5,6^

The OOPAAI is described in detail in Utinger et al.^30^ and Section S1.4 in the Supporting Information. Particles are continuously measured using a commercial particle-into-liquid sampler (PILS, Brechtel, USA) at a flow rate of 16 L min^–1^ and immediately sampled into a wash flow containing 200 μM ascorbic acid (AA), where the particle AA mixture is reacted for 10 min at 37 °C in a heated bath. The OOPAAI measures OP_AA_ by quantifying the formation of dehydroascorbic acid (DHA), the dominant oxidation product of ascorbic acid (AA), by reacting DHA with *o*-phenylenediamine (OPDA), forming the fluorescent product 3-(1,2-dihydroxyethyl)-fluoro[3,4-*b*]quinoxalin-1-one (DFQ). The concentration of DFQ is then quantified using fluorescence spectroscopy (λ_ex_ = 365 nm and λ_em_ = 430 nm). The OOPAAI is calibrated using known concentrations of DHA at pH 6.8, and hence the OP_AA_ here is then expressed in terms of nanomoles of DHA per unit volume (m^–3^) or unit mass (μg^–1^). For comparison with online measurements, BSOA and NSOA particles were collected on 47 mm Teflon filters for 1 h at a flow rate of 10 LPM. SOA filter samples were extracted within 1 h of collection for as close as practically possible comparison with direct online measurements. For each SOA comparison, online filters were collected and analyzed on the same day as the online OPROSI or OOPAAI measurement. Filters were extracted and analyzed using the DCFH and AA assays under the same chemical conditions for online measurements using protocols described in full in Campbell et al.^9^

### 2.3 Quantification of OP_OH_

Hydroxyl radical production (OP_OH_) was quantified using the terephthalate probe (TA).^14^ TA reacts selectively with OH to produce the highly fluorescent product 2-hydroxyterepthalate (hTA), which is then detected at λ_ex_ = 320 and λ_em_ = 420 nm. A 325 nm peak emission LED (M325F4, Thorlabs) is coupled to a cuvette cell (CVH100), using quartz cuvettes to ensure efficient UV transmission and a QEpro (Ocean insight) high precision spectrometer to facilitate fluorescence detection. SOA samples were extracted into 10 mM TA at pH 6.8, in HEPES buffer containing 200 μM AA at particle concentrations equivalent to those sampled using the OPROSI and OOPAAI. SOA produced using the OCU was collected on filters prior to OP_OH_ analysis. Equivalent concentrations of Fe(II)SO_4_ and Cu(II)SO_4_ that were sampled by the OOPAAI and OOPROSI experiments were added to SOA filter samples. Detailed descriptions of filter collection methods are given in Section S1.2 in the Supporting Information.

### 2.4 Chemical Kinetics Model Development

The model describing iron, copper, ROS, hydroperoxide, and quinone chemistry in the presence of AA is presented in Table S2 in the Supporting Information. It includes 137 individual reactions and builds on the previous model presented by Shen et al.,^31^ which describes the redox chemistry of ascorbic acid (AA) with ROS, Fe(II)/Fe(III), and Cu(I)/Cu(II). It also includes reactions describing the AA assay measuring DHA formation (OP_AA_) as described in Campbell et al., which is used in this work.^29^ The kinetic model uses a catalytic mechanism to describe the oxidation chemistry of ascorbic acid in the presence of Fe(II), Fe(III), and Cu(II), as opposed to a redox reaction. While recent evidence has demonstrated that the redox reaction may play a role, based on the observation of the ascorbyl radical by Wei et al.,^35^ there is convincing evidence in the literature which also supports the catalytic reaction. In addition, the catalytic reaction predicts DHA formation reaonably well in Shen et al.,^31^ while the redox reaction underpredicted DHA formation. Sensitivity tests were previously performed including both the redox and catalytic tests, which again lends support to the catalytic mechanism. Detailed discussion of the model mechanism can be found in Shen et al.^31^

In this study, we further developed the model by adding the following reactions: chemistry describing the reaction of naphthoquinones with AA, ROS, Fe(III), and Cu(II), as well as organic peroxide chemistry, TA probe reactions with OH, iron-HULIS complexation and subsequent reactions, based on the data presented in Gonzalez et al.,^14^ as well as HEPES and phosphate buffer chemistry (Table S2). Reactions and rate constants were synthesized from the literature and referenced appropriately in Table S2. The kinetic model was solved using the Kinetics Pre-Processor (KPP) version 2.2.3,^36^ utilizing the Rosenbrock solver and gFortran compiler.

The model was run using the experimental conditions in the OOPAAI model for each individual experiment. pH was initially set at pH 7 and then equilibrated to pH 6.8 by using 10 mM HEPES buffer in the model input (R130–131, Table S2). The model was run at pH 6.8 for 10 min and then at pH 2 for 2 min to simulate the experimental conditions in the OOPAAI as described in Shen et al.^31^ and Campbell et al.^29^ The majority of the rate constants presented in Table S2 are determined at room temperature, whereas measurements using OOPAAI are conducted at 37 °C, which may introduce uncertainty regarding model calculations.

For the model data presented in this study, some of the chemistry is well established, including much of the ROS chemistry, acid–base equilibria, inorganic iron chemistry, and probe and buffer chemistry. There are several general sources of error and uncertainty for the set of reactions in Table S1 in addition to the specific uncertainties described above. These include errors in the rate constants, which range from a few percent to a factor of 10 or more. In some cases, reaction stoichiometries and product distributions are also uncertain.

## 3 Results and Discussion

### 3.1 Comparison of Online and Offline Measurements of SOA OP

**Figure 1:**
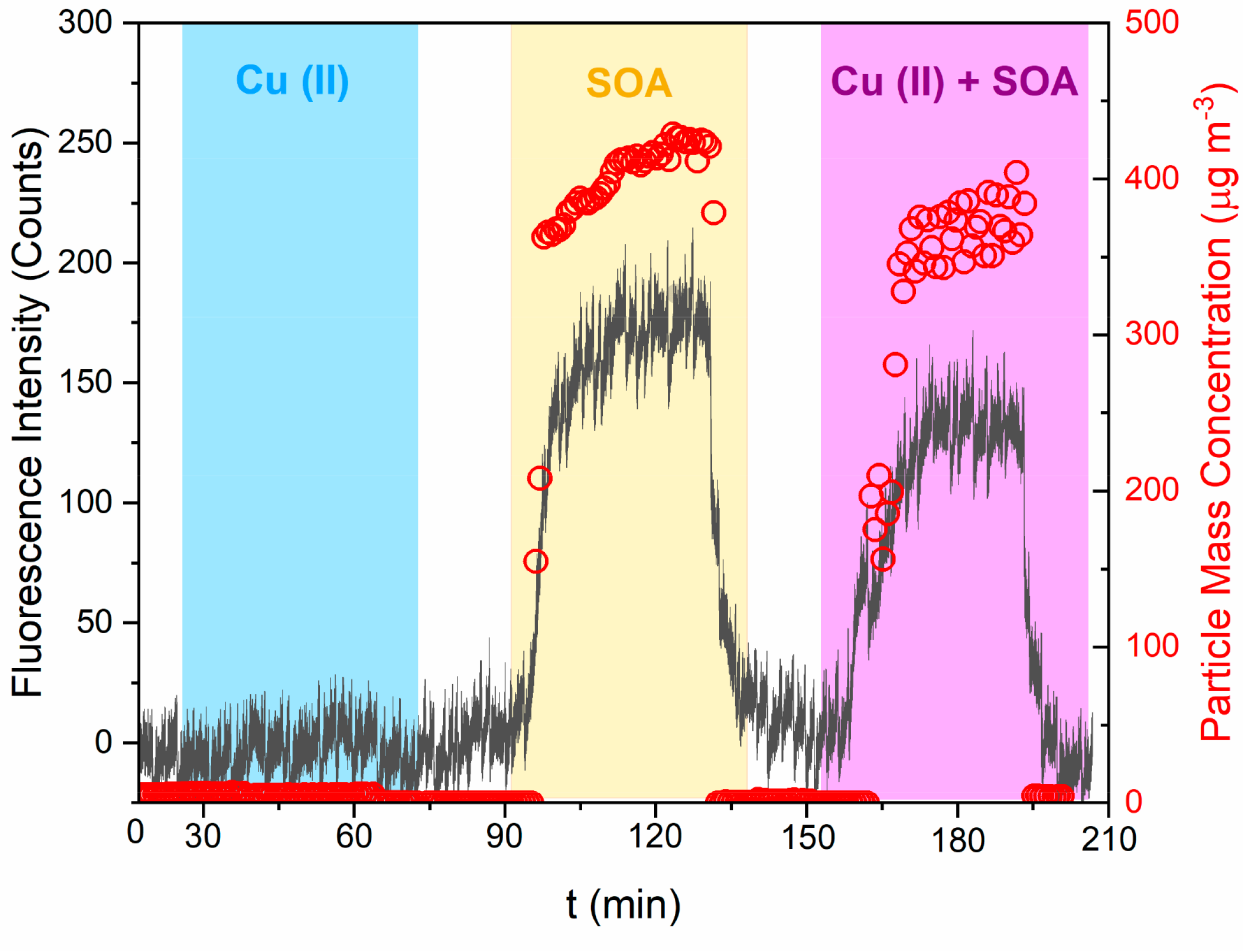
Representative time-corrected online data illustrating the response of the OPROSI to Cu(II) (blue), ß-pinene SOA (yellow), and a mixture of Cu(II) and ß-pinene SOA (purple).

Using the experimental setup described in Figure S1, online particle-bound ROS_DCFH_ and OP_AA_ were quantified for ß-pinene-derived SOA (BSOA), naphthalene-derived SOA (NSOA), and Fe(II) and Cu(II) particles. A representative plot illustrating the online response of the OPROSI as a function of Cu(II), BSOA, and Cu(II) + BSOA particle mass is presented in Figure 1. Experiments in this study are performed by quantifying the individual ROS_DCFH_, OP_AA_, and OP_OH_ of metal seed particles and SOA and then quantifying OP for metal seed seeds coated with both BSOA and NSOA. Particles are well mixed as evidenced by the growth of particle size distribution, where one mode is observed for SOA + metal mixtures produced in the OCU (Figure S2).

**Figure 2:**
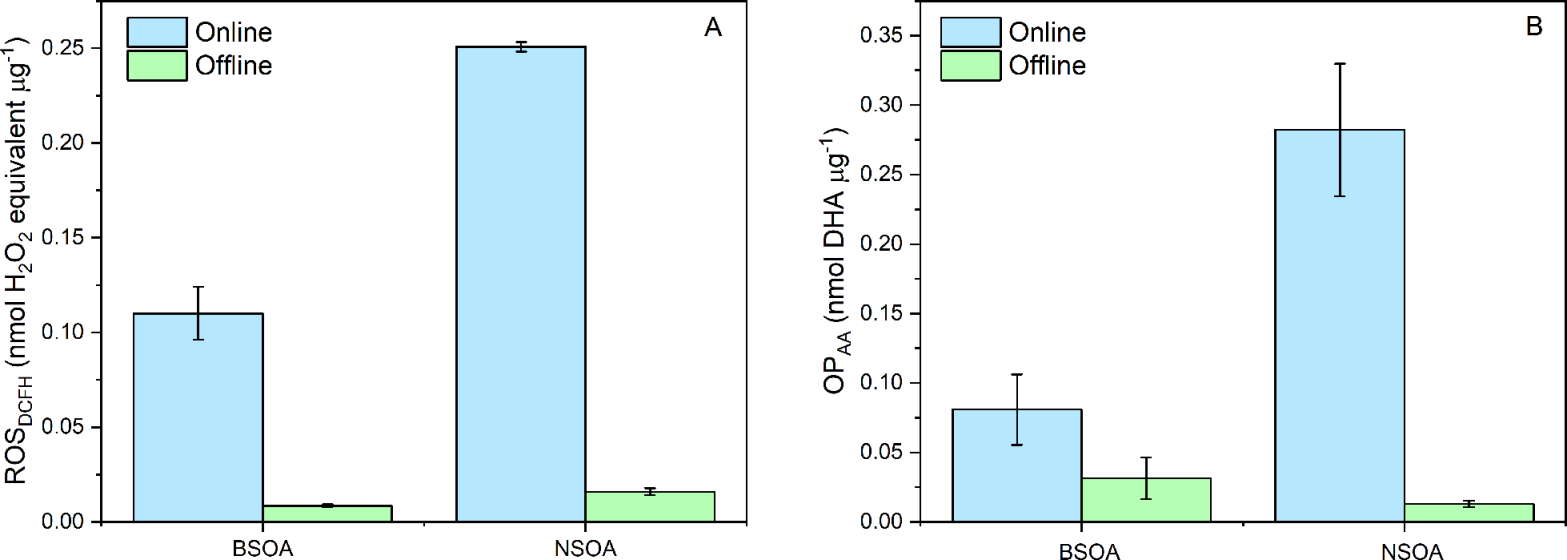
Comparison of both online and offline mass-normalized OP responses for BSOA and NSOA for (A) ROS_DCFH_ and (B) OP_AA_. Error bars represent the standard deviation observed over 3 experimental repeats.

A comparison between online and filter-based offline ROS_DCFH_ and OP_AA_ measurements is presented in Figure 2. Here, we clearly show that offline-based methods substantially underestimate the ROS_DCFH_ and OP_AA_ of SOA. As shown in Figure 2A, the intrinsic mass-normalized ROS_DCFH_ activity of both BSOA and NSOA is substantially lower than online methods, with offline values of 0.085 ± 0.007 nmol H_2_O_2_ equivalent μg^–1^ and 0.015 ± 0.002 nmol H_2_O_2_ equivalent μg^–1^, respectively. In comparison, online measurements of ROS_DCFH_ were 0.11 ± 0.02 nmol of H_2_O_2_ equivalent μg^–1^ and 0.25 ± 0.014 nmol of H_2_O_2_ equivalent μg^–1^ for BSOA and NSOA, respectively. This equates to a 93% decrease in BSOA ROS_DCFH_ and a 94% decrease in NSOA ROS_DCFH_ activity of particles collected on filters compared to those from online methods. This is in good agreement with previous studies from our group by Fuller et al.^5^ and Zhang et al.,^27^ who also observed >90% decrease in particle-bound ROS comparing online and offline filter based ROS_DCFH_ measurements.

In addition, we present the first comparison of online and offline filter-based measurements of SOA OP_AA_ using the OOPAAI (Figure 2B). Similar to ROS_DCFH_, BSOA and NSOA particle OP_AA_ is substantially underestimated using offline filter measurements when comparing to online OP_AA_.For BSOA, online OP_AA_ was measured to be 0.08 ± 0.02 nmol DHA μg^–1^ compared to offline 0.034 ± 0.015 nmol DHA μg^–1^, and for NSOA an online OP_AA_ of 0.28 ± 0.05 nmol DHA μg^–1^ compared to 0.012 ± 0.002 nmol DHA μg^–1^ for offline. This is equivalent to ∼67% and ∼95% reductions in filter OP_AA_ activity. These results demonstrate specifically that decomposition of labile organic compounds present in SOA, such as ROOH/ROOR, and potentially quinones leads to a reduction in ROS_DCFH_ and OP_AA_ activity when measured using a traditional offline filter-based method. This emphasizes the importance of rapid, direct-to-reagent (<1 min) measurement methods for robust quantification of particle ROS and OP activity of organic aerosol. Therefore, in order to fully determine the interplay of transiton metals and SOA, where Fenton-like reactions play a crucial role, online methods which fully capture aerosol chemistry occurring on fast time scales are required.

### 3.2 Online ROS_DCFH_ and OP_AA_ of BSOA, NSOA, Fe(II), and Cu(II)

#### 3.2.1 ROS_DCFH_

**Figure 3:**
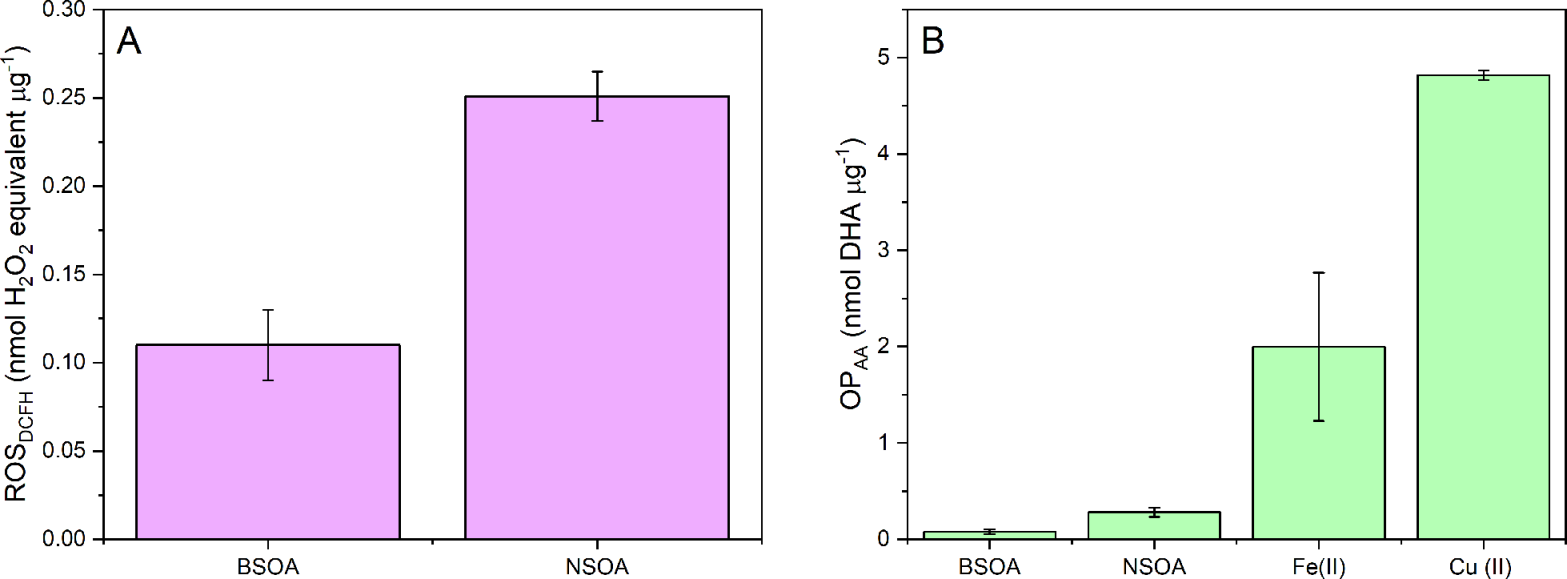
(A) ROS_DCFH_ and (B) OP_AA_ values measured for BSOA, NSOA Fe(II), and Cu(II). Error bars represent the standard deviation observed over three experimental repeats. Note that for Cu(II) and Fe(II), no ROS_DCFH_ signal was observed.

ROS_DCFH_ and OP_AA_ for individual BSOA, NSOA, and transition metals are summarized in Figure 3. Representative online data are presented in Figure 1. NSOA shows almost a factor of 2 greater ROS_DCFH_ compared to BSOA, with an ROS_DCFH_ of 0.25 ± 0.01 nmol H_2_O_2_ equivalent μg^–1^ and 0.11 ± 0.02 nmol H_2_O_2_ equivalent μg^–1^, respectively (Figure 3A). This observation is in good agreement with our previous study by Zhang et al. investigating NSOA and BSOA ROS_DCFH_ using the OPROSI.^27^ ROS_DCFH_ observed previously for limonene and oleic acid SOA were 0.4 and 0.58 nmol H_2_O_2_ equivalent μg^–1^, respectively.^5,37^ Therefore, SOA derived from different precursors of both biogenic and anthropogenic origin have substantially different ROS_DCFH_, with up to a factor ∼3 difference depending on the SOA precursor. No online ROS_DCFH_ signal was observed when nebulized Cu(II) or Fe(II) particles were sampled with the OPROSI, as the DCFH assay is predominantly sensitive to hydrogen peroxide and organic peroxides.^5,6^

#### 3.2.2 OP_AA_

OP_AA_ values, expressed in nmol DHA μg^–1^, are presented in Figure 3B. As is the case with ROS_DCFH_, higher intrinsic OP_AA_ is observed for NSOA (0.28 ± 0.05 nmol DHA μg^–1^) compared to BSOA (0.08 ± 0.02 nmol DHA μg^–1^). Increased NSOA activity for OP_AA_ may be due to the presence of naphthoquinones in NSOA. Experiments were performed to determine OP_AA_ to a range of individual compounds, including commercially available organic peroxides, and naphthoquinones which have been previously detected in NSOA^12^ are presented in Figure S5. 1,2-Napthoquionone (1,2-NQN), shows greater OP_AA_ compared to equivalent concentrations of a range of commercially available organic peroxides and is also more OP_AA_ active compared to equivalent concentrations of Fe(II) and Cu(II), highlighting that naphthoquinones may be key drivers of NSOA OP_AA_. Redox-active transition metals, particularly Fe(II) (1.99 ± 0.76 nmol DHA μg^–1^) and Cu(II) (4.81 ± 0.02 nmol DHA μg^–1^), exhibit an order of magnitude higher OP_AA_ compared to BSOA and NSOA. The sensitivity of the AA assay toward redox-active transition metals, in particular Fe(II) and Cu(II), has been well documented in previous studies.^9,31^ A recent study by Shen et al.^31^ has suggested that redox-active transition metals, specifically Fe(III) and Cu(II), catalytically react with AA (and ascorbate, AH^–^, the dominant form of AA at pH 6.8). This direct oxidation of AA/AH^–^ by transition metals such as Fe(III) (produced in these experiments from Fe(II) oxidation) and Cu(II) results in the formation of DHA through the following reactions:^31^
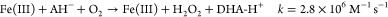
R1
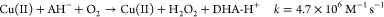
R2Therefore, given the higher rate constant in eq R2, enhanced direct DHA production is expected in the case of Cu(II) compared to Fe(II). In addition, according to model runs using visual MINTEQ (v.3.1) (Figures S6 and S7), Fe(III) will exist almost entirely as the relatively insoluble form Fe(OH)_2_^+^ at pH 6.8, which may further limit its ability to participate in eq R1 compared to Cu(II).

### 3.3 Influence of Fe(II) and Cu(II) on ROS_DCFH_ of NSOA and BSOA

**Figure 4:**
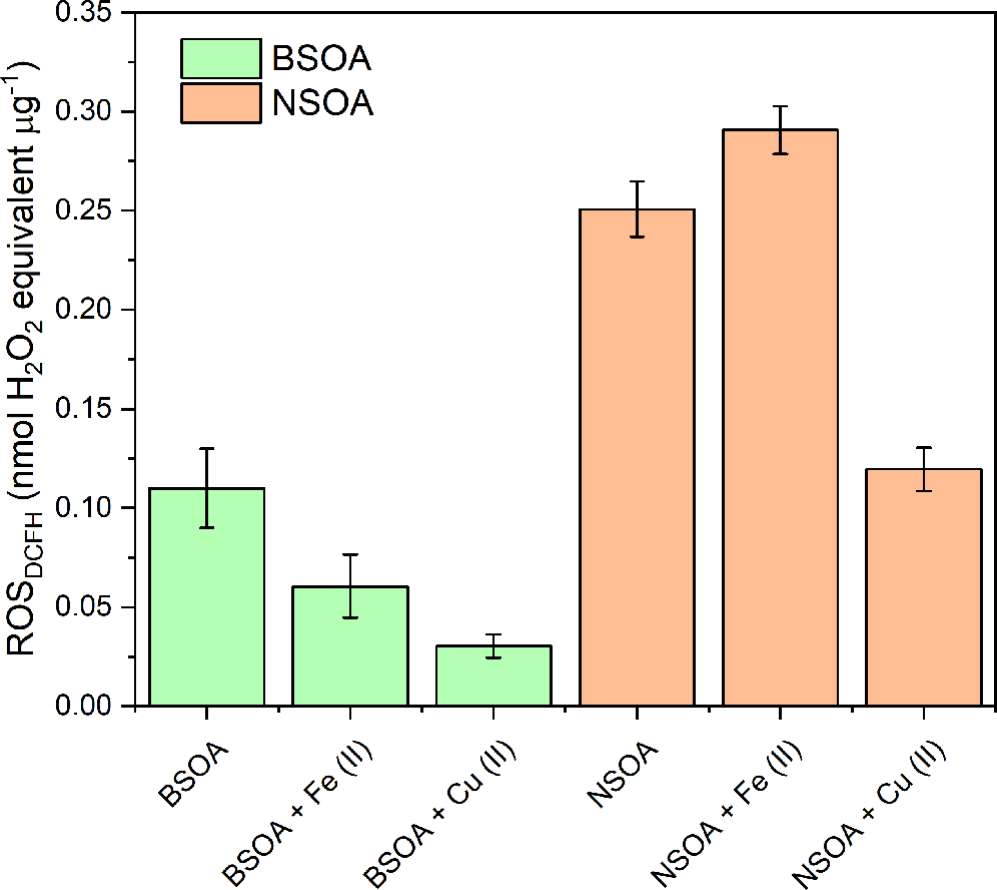
ROS_DCFH_ for pure BSOA (green) and NSOA (orange) and mixtures of BSOA and NSOA with Fe(II) and Cu(II) seed particles. Error bars represent the standard deviation over four experimental repeats (BSOA and NSOA) and average signal observed over a 1 h continuous online sampling period for SOA–metal mixtures.

We investigated the influence of mixing Fe(II) and Cu(II) seed particles with BSOA and NSOA on ROS_DCFH_ and OP_AA_ using the OPROSI and OOPAAI, respectively. For all measurements, the two instruments were run in parallel using the experimental apparatus described in Figure S1. Comparison of ROS_DCFH_ values for BSOA and NSOA mixed with Fe(II) and Cu(II) seeds is presented in Figure 4.

For both BSOA and NSOA, the ROS_DCFH_ activity generally decreases when both Fe(II) and Cu(II) seed particles are present. Compared to BSOA only (0.11 ± 0.02 nmol H_2_O_2_ equivalent μg^–1^), the intrinsic mass-normalized ROS_DCFH_ of BSOA + Cu(II) and BSOA + Fe(II) decreases to 0.03 ± 0.006 and 0.06 ± 0.015 H_2_O_2_ equivalent μg^–1^, respectively. The DCFH assay predominantly measures H_2_O_2_, organic hydroperoxides, and organic peroxides.^5,6^ BSOA has been shown to be particularly rich in ROOH/ROOR.^38^ Tong et al.^17^ measured the yield of organic peroxides for BSOA and NSOA as 42 ± 24% and 19 ± 7%, respectively. In addition, they reported mass-normalized H_2_O_2_ production from BSOA and NSOA in H_2_O as 5.47 ± 1.24 and 0.67 ± 0.66 ng/μg, respectively, and in SLF of 4.52 ± 0.08, 16.3 ± 4.4 ng/μg, respectively. It should be noted that the referenced studies by Tong et al.^17,18^ use a filter-based approach and likely characterize long-lived peroxides. As evidenced by Figure 2, the online method captures the chemistry of reactive (and hence relatively short-lived) and long-lived peroxides, which contribute a substantial fraction of ROS_DCFH_. They observe a difference in BSOA and NSOA peroxide yields that contradict our findings and those of Zhang et al.,^27^ but this is likely due to the different chemistry of short-lived peroxides. Therefore, the observed decrease in ROS_DCFH_ for BSOA and NSOA in the presence of Fe(II) and Cu(II) may well be due to the enhanced decomposition of H_2_O_2_, as well as both short-lived and long lived organic peroxides in SOA by Fenton-like reactions with Fe(II) and Cu(II).

We tested the ROS_DCFH_ activity of a range of peroxide standards including cumene hydroperoxide, benzoyl peroxide, and *tert*-butyl hydroperoxide, commercially available peroxides that act as surrogates for peroxides expected in BSOA and NSOA, in addition to mixtures of these peroxides with Fe(II) and Cu(II) (Figure S4). A decrease in ROS_DCFH_ is observed when these organic peroxides are mixed with Fe(II) and Cu(II), demonstrating that Fe(II) and Cu(II) can also decompose a range of organic peroxides, reducing ROS_DCFH_. Interestingly, a greater decrease in ROS_DCFH_ is observed when peroxides are mixed with Cu(II) compared with Fe(II), in agreement with our observations for BSOA + Cu(II) (Figure 4). Cu(II) reactions with H_2_O_2_ (*k* = 480 M^–1^ s^–1^)^39^ have been suggested to be faster than the Fenton reaction between Fe(II) (*k* = 55 M^–1^ s^–1^)^42^ and H_2_O_2_, proceeding as follows:
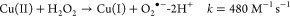
R3
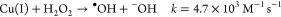
R4To validate the above mechanisms, we quantified ^•^OH produced from the Cu(II) + H_2_O_2_ reaction and compared it to a simplified kinetic model (Table S2) which predicts ^•^OH formation based on eqs R3 and R4 (Figure S8). We observe reasonably good agreement between the formation of ^•^OH from Cu(II) and H_2_O_2_ and the kinetic model over time, highlighting the feasibility of eqs R3 and R4. Therefore, enhanced particle-bound peroxide decomposition via Cu(II) chemistry (liberating O_2_^•–^ and ^•^OH) could explain the enhanced decrease of BSOA and NSOA ROS_DCFH_ of Cu(II) compared to Fe(II). There are limited literature data regarding the reaction of Cu(II) and Fe(II) Fenton-like reactions with larger organic peroxides or hydroperoxides. Fang et al.^26^ demonstrated that isoprene hydroxy hydroperoxides (ISOPOOH), prevalent in isoprene-derived SOA, is rapidly consumed by Fe(II), at a rate substantially greater than for the Fenton reaction with H_2_O_2_ (*k* ∼ 4 × 10^4^ M^–1^ s^–1^ compared to *k* =55 M^–1^ s^–1^).^26,42^ Thus, some organic peroxides present in BSOA may also exhibit similar enhanced Fenton-like reactivity toward Fe(II). It has also been demonstrated that the reaction of Fe(II) with organic peracids, which are common labile peroxides in BSOA,^40^ is potentially rapid; for example, the rate constant for Fe(II) plus peracetic acid (PAA) is 5 × 10^4^ M^–1^ s^–1^^41^ at circumneutral pH compared to that of Fe(II) + H_2_O_2_ (55 M^–1^ s^–1^),^42^ likely due to the lower Δ*G*_f_ associated with Fe(II) + PAA (−299.8) compared to Fe(II) + H_2_O_2_ (−118.5)^41^ and reduced bond energy of O–OH for PAA (88.4 kcal mol^–1^) compared to H_2_O_2_ (90.4 kcal mol^–1^).^41,43^ Thus, the Fe(II) + PAA Fenton-like reaction is more favorable compared to Fe(II) + H_2_O_2,_ a process which could also be at play here.^44−46^ In addition, Wei et al.^35^ demonstrated that iron-facilitated reactions with organic hydroperoxides in the presence of isoprene SOA produce substantially more radical species in both aqueous extracts and SLF.^35^ Given the higher rate constant between Cu(II) and H_2_O_2_, it is plausible that enhanced degradation of ROOR/ROOH in the presence of Cu(I) and Cu(II) would also be observed, thus resulting in an enhanced decrease of particle-bound peroxides compared to Fe(II).

Furthermore, NSOA formed via photooxidation is known to produce quinones and semiquinone radicals, which when extracted in water can react with O_2_ to form superoxide (O_2_^.–^) and therefore potentially produce more ROS compared to BSOA.^47^ Similar to BSOA, the largest decrease in NSOA ROS_DCFH_ is also observed when NSOA and Cu(II) are mixed (Figure 4), likely due to the enhanced destruction of both organic peroxides and H_2_O_2_ produced from NSOA by Cu(II) and Cu (I). Wang et al.^21^ demonstrated using ^1^H NMR that Cu(II) complexes with components present in photooxidized NSOA, with dominant chemical components such as 1,2 naphthoquinone or 2,3-dihydroxynaphthalene, resulting in a decrease in DTT activity due to limited redox chemistry as a result of Cu(II) complexation.^21^ This phenomenon may explain the decrease in ROS_DCFH_ observed here, where the ability of quinones and semiquinones to produce H_2_O_2_ is reduced as a result of Cu(II) complexation. Interestingly, a modest increase in ROS_DCFH_ is observed when Fe(II) is mixed with NSOA. There are limited studies investigating the interaction of NSOA components with Fe(II) and Fe (III) directly. However, a few studies have investigated the chemistry of quinones and hydroquinones with Fe(II)/Fe(III); Li et al.^48^ showed enhanced OH production from anthraquinone and Fe(II), likely due to enhanced redox cycling of semiquinone chemistry.^48^ Jiang et al.^49^ demonstrated that Fe(III) interacts with 1,4-hydroquinone, producing semiquinone radicals, which can in turn produce ROS and H_2_O_2_, although these measurements were performed under more acidic conditions (pH 5) than this study. In addition, Zanca et al.^50^ measured the yield of humic-like substances (HULIS) in NSOA formed in an aerosol flow reactor to be around 30%.^50^ Complexation of HULIS with Fe has been shown to enhance the redox chemistry of Fe(II),^20^ another process which may explain the enhanced ROS_DCFH_ of NSOA in the presence of Fe(II).

### 3.4 Synergistic and Antagonistic Effects of Transition Metals on OP_AA_ and OP_OH_

**Figure 5:**
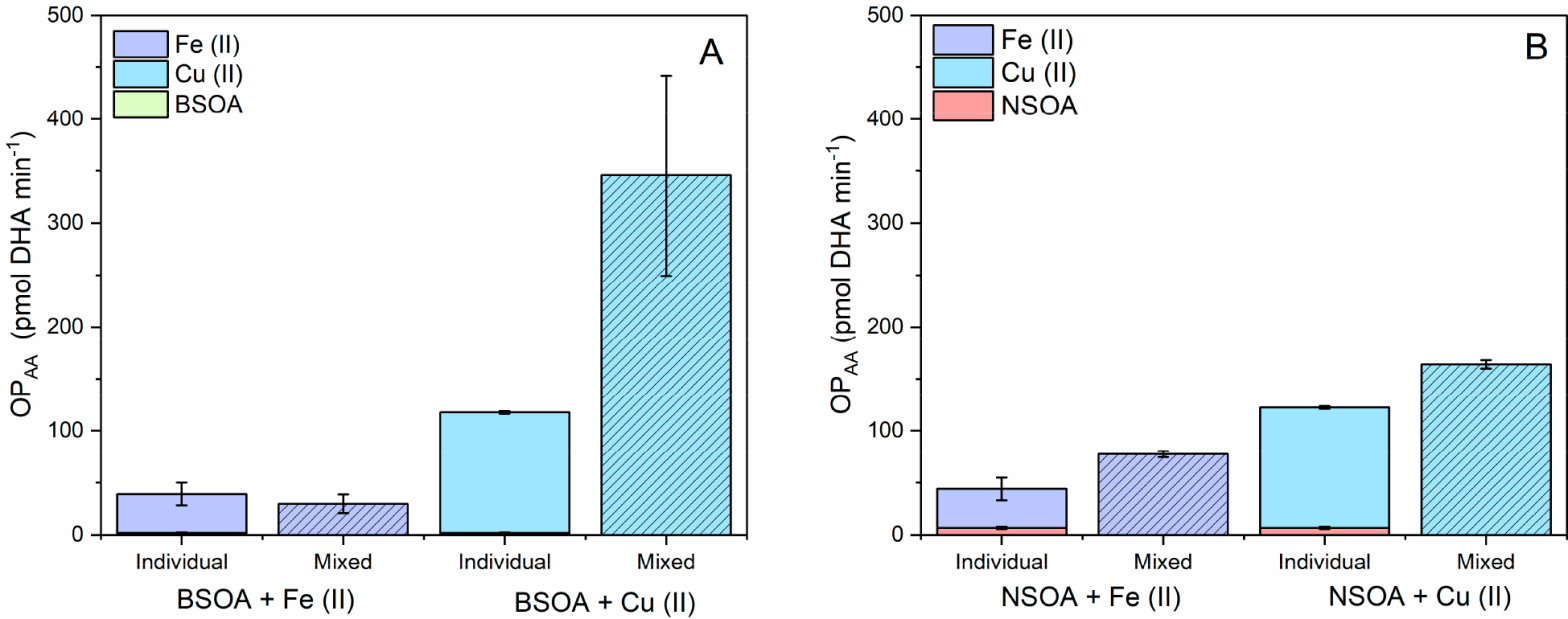
OP_AA_ for (A) BSOA and (B) NSOA, plus Fe(II) and Cu(II) seed particles, comparing the sum of the individual OP_AA_ responses of BSOA, NSOA, Fe(II), and Cu(II) with mixtures of SOA and metal seeds. Note that OP_AA_ for “individual” BSOA in (A) the bars are barely visible due to their small response compared to the respective values for Fe(II) and Cu(II) (see Figure 3B). Error bars represent the standard deviation of the online signal observed over 1 h sampling.

In addition to online ROS_DCFH_ measurements, online OP_AA_ measurements of Fe(II) and Cu(II) mixed with BSOA and NSOA were performed. The results are presented in Figure 5, which shows the relative increase or decrease in OP_AA_ when a transition metal and SOA are mixed relative to the sum of their individual OP_AA_. Note that these values are not mass normalized, due to the much higher intrinsic OP_AA_ activity of Cu(II) and Fe(II) per mass compared to BSOA and NSOA (Figure 3). The comparison of individual components (i.e metals and SOA) with the mixture of metals and SOA is still possible because the same amounts of metal and SOA were considered for each condition.

There are clear synergistic and antagonistic effects based on the transition metal and the type of SOA. Suppression of BSOA OP_AA_ is observed when BSOA is mixed with Fe(II) (Figure 5A), decreasing from 39.4 pmol DHA min^–1^ (combined sum of OP_AA_ for Fe(II) and BSOA, Figure 5A) to 29.7 pmol min^–1^ when mixed. Complexation of Fe(II) with chemical components common in BSOA, such as carboxylic acids and aldehydes, may limit the redox activity of Fe(II) via complexation,^51^ as well as limiting the ability of Fe(III) to directly oxidize AA to form DHA.^31^ In contrast, a substantial increase in OP_AA_ is observed when Cu(II) seed particles are mixed with BSOA (345 pmol DHA min^–1^) relative to the sum of the individual OP_AA_ of BSOA and Cu(II) (117.4 pmol DHA min^–1^). This coincides with the greatest decrease in online ROS_DCFH_ (Figure 4), where a decrease in ROS_DCFH_ suggests that there is a larger decrease in peroxide content in BSOA when Cu(II) is present compared to Fe(II). The reaction of Cu(II) with ROOH/ROOR present in BSOA may then produce hydroxyl radicals or other organic radicals via Fenton-like chemistry, potentially leading to a more pronounced increase in the level of DHA formation (i.e., an increase in OP_AA_). Enhanced AA loss and OH production have previously been observed for mixtures of Cu(II), H_2_O_2_, and AA.^52,24^ This may indicate that the reaction of Cu(II)/Cu (I) and ROOH/ROOR in the presence of AA may enhance OH production and DHA formation, increasing OP_AA_. AA, and ascorbate (AH^–^), the deprotonated form of AA, which will be the dominant form under the experimental conditions here (pH 7.4), is known to be relatively unreactive toward peroxides^53^ and may be even less sensitive to larger organic peroxides and hydroperoxides with increased steric hindrance. Therefore, the rapid conversion of peroxides to hydroxyl or alkoxyl radicals by Cu(II) in SOA, which oxidize AH^–^ much more rapidly than peroxides, given the rate constant for AH^–^ + H_2_O_2_ (*k* ∼ 1.6 × 10^2^ M^–1^ s^–1^)^53^ compared to that of AH^–^ + OH (*k* = 7.9 × 10^9^ M^–1^ s^–1^), likely increases OP_AA_. Cu(II) complexation may play an additional role here in enhancing DHA production and OH production. Yan et al.^54^ demonstrated that Cu(II) mixed with water-soluble organic carbon (WSOC) enhanced OH production and AA loss, and Lin et al.^51^ showed that mixtures of Cu(II) and complexing ligands such as citrate, malonate, and oxalate also enhance OH production and AA loss. Therefore, the interaction of the BSOA components and Cu(II) may potentially explain the observed enhancement of OP_AA_ for BSOA + Cu (II).

For NSOA, synergistic enhancements of OP_AA_ are observed for NSOA + Cu(II) and Fe(II). The greatest % enhancement is observed for NSOA + Fe(II), from 43.8 to 77.3 pmol min^–1^. This could be driven by interactions with quinones or complexation with HULIS-like molecules formed during naphthalene photooxidation, which contain a range of functionalized aromatic moieties.^47^ Enhanced OP_AA_ is also observed when NSOA is mixed with Cu(II), increasing from 121.2 pmol of DHA min^–1^ to 163.9 pmol of DHA min^–1^. Enhanced decomposition of H_2_O_2_, which has been shown to be produced by NSOA upon aqueous extraction,^17^ by Cu(II) could increase OH production and hence OP_AA_. In addition, the presence of organic ligands in NSOA such as naphthoquinones, hydroquinones, or HULIS-like molecules in NSOA could enhance the redox potential of the metals themselves. For instance, this could enhance their direct oxidation pathways leading to DHA formation and AA degradation and hence an increased OP_AA_.^31^

For both BSOA and NSOA, we hypothesize that transition metals participate in Fenton-like chemistry with particle-phase peroxides, either formed during particle formation via VOC photooxidation or with hydrogen peroxide which has been shown to be formed during BSOA and NSOA extraction in aqueous media.^18^ The reaction of metals with peroxides liberates more reactive ROS species such as OH and organic radicals, which leads to enhanced DHA formation increasing OP_AA_.

**Figure 6:**
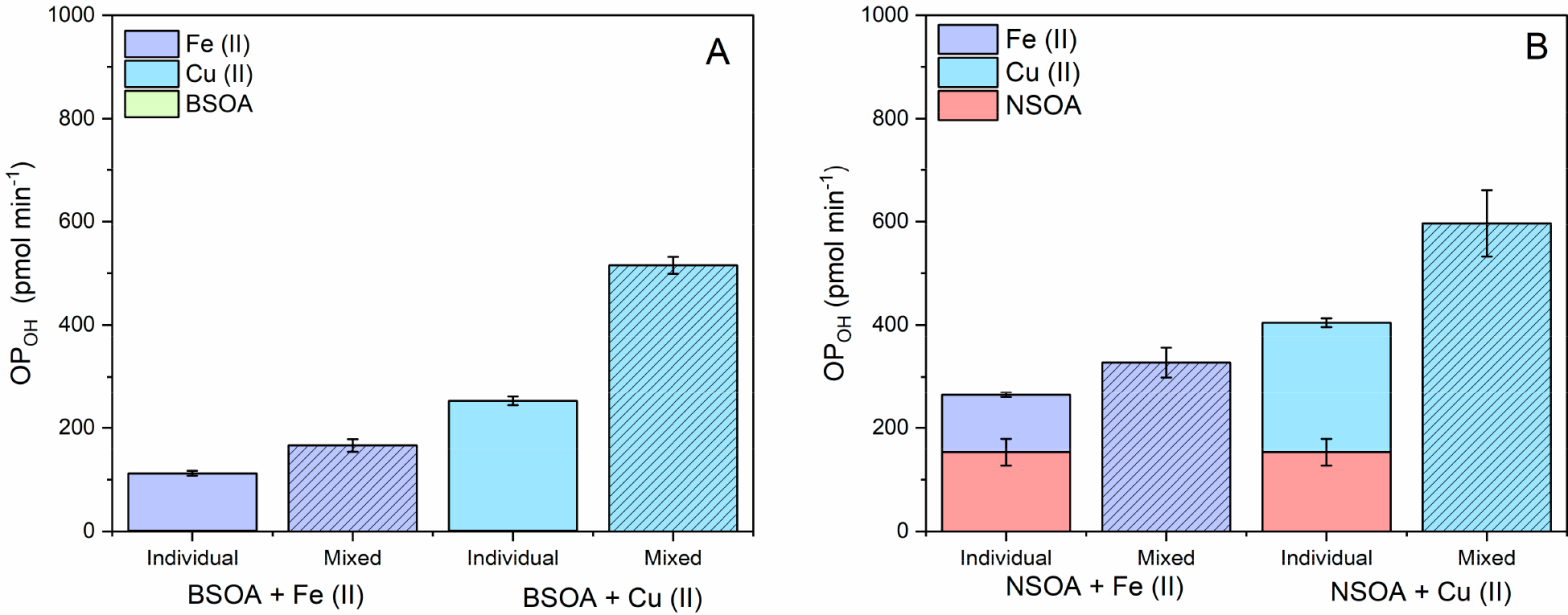
OP_OH_ measured for individual components and mixtures of (A) BSOA with Fe(II) and Cu(II) and (B) NSOA with Fe(II) and Cu(II), all in the presence of 200 μM AA. Hatched lines indicate experiments where the SOA and metal particles are mixed. Note that BSOA only OP_OH_ values are substantially lower (0.7 ± 0.06 pmol min^–1^) than others plotted in Figure 6. OP_OH_ experiments were performed at metal and SOA mass concentrations equivalent to those of OP_AA_ measurements. Error bars represent the standard deviation observed over three experimental repeats.

To test this, we also measured OP_OH_ from mixtures of BSOA and NSOA with Fe(II) and Cu(II)) all in the presence of AA. These experiments were conducted for the same particle concentrations, AA concentrations, and metal/SOA mixing ratios as the OOPAAI measurements for each condition discussed earlier for a direct comparison, the results of which are presented in Figure 6A (BSOA) and Figure 6B (NSOA).

OP_OH_ measurements are in broad agreement with the observed OP_AA_ values. As was the case with OP_AA_, we observe a synergistic enhancement of OP_OH_ for both BSOA and NSOA in the presence of transition metals, notably, the redox-active Fe(II) and Cu(II). OP_OH_ for BSOA is substantially lower than that for NSOA, 0.7 ± 0.06 pmol min^–1^ compared to 153 ± 25 pmol min^–1^, respectively. This result is in broad agreement with those of ROS_DCFH_ and OP_AA_ for BSOA and NSOA (Figure 3). For BSOA, addition of Fe(II) and Cu(II) synergistically enhances OH production compared to the sum of their individual OH production rates in the presence of AA, with BSOA + Fe(II) + AA and BSOA + Cu(II) + AA OH production rates of 186 ± 0.13 and 515 ± 16 pmol min^–1^ respectively. Higher OP_OH_ production is also observed for NSOA + Fe(II) and Cu(II), with 327 ± 28 and 596 ± 64 pmol min^–1^ respectively. OP_OH_ measurements are in broad agreement with OP_AA_ measurements, as well with decrease in ROS_DCFH_, which we hypothesize is likely due to decomposition of H_2_O_2_ and ROOH/ROOR from SOA by transition metals upon aqueous extraction, increasing OP_OH_.

### 3.5 Kinetic Modeling of OP_AA_

**Figure 7:**
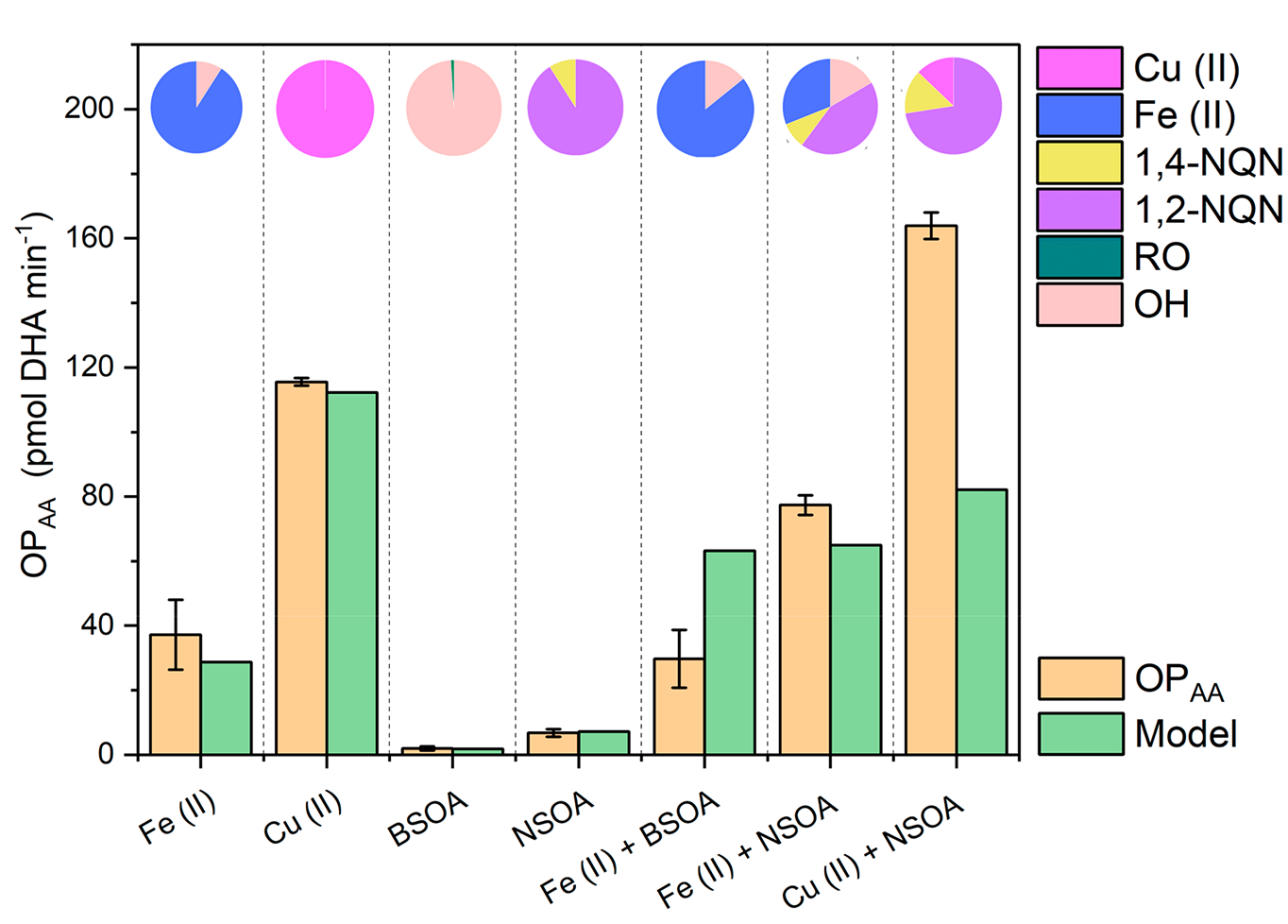
Comparison of OP_AA_ measurements (orange bars) with kinetic model results (green bars). Pie charts indicate relative contributions of key redox-active species in the model toward DHA formation and hence OP_AA_.

Modeling results and measurement data for DHA formation from AA oxidation (OP_AA_) from BSOA, NSOA, Fe(II), Cu(II), and SOA–metal mixtures are presented in Figure 7. In addition, pie charts within Figure 7 for each experimental condition indicate the contribution of key reactive species toward modeled OP_AA_. Overall, the model is in relatively good agreement regarding measured OP_AA_ (i.e., DHA formation) especially for Fe(II) and Cu(II), as well as BSOA and NSOA (Figure 7).

#### 3.5.1 Metals + AA

The model suggests that Fenton-like chemistry involving Fe(II)/Cu(I) + H_2_O_2_ → OH + OH^–^ only plays a minor role promoting DHA formation, consistent with the study by Shen et al.^31^ Instead, direct reactions of Fe(III), formed from Fe(II) oxidation, and Cu(II) with AH^–^, the dominant deprotonated form at pH 7.4, are the dominant pathways for DHA formation (∼92%, ∼99%, respectively, Figure 7) via the catalytic reactions of ascorbate (AH^–^) (eqs R1 and R2) under these reaction conditions.^31^

#### 3.5.2 BSOA + AA

Production of DHA from BSOA in the model comes predominantly from OH formation from the homolysis of organic peroxides (ROOH), producing OH and the alkoxyl radical (RO):^18^
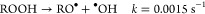
R5OP_AA_ is particularly sensitive to the combination of the *k* for eqR5 and the assumed concentration of ROOH in BSOA. OP_AA_ is well predicted by the model when considering the estimated first order rate constant^18^*k* = 0.0015 s^–1^ and an ROOH yield of ∼80% (assuming an average molar mass of 205 g mol^–1^ for BSOA), which is within the range of reported ROOH yields of 30–90% previously observed in BSOA.^38^ RO contributes substantially less to DHA formation in the BSOA model, despite being formed in equal amounts to OH. The rate constant of AA/AH^–^ + RO (*k* = 1 × 10^4^ M^–1^ s^–1^)^18^ is orders of magnitude lower compared to that of AA/AH^–^ + OH (*k* = 7.9 × 10^9^ to 1.1 × 10^10^ M^–1^ s^–1^).^55,56^ This is consistent with EPR data from Wei et al.^35^ Using spin-trapping coupled to EPR, Wei et al. demonstrated that the composition of radical species substantially changes when isoprene SOA and Fe(II) were mixed in water and SLF. They observed a near total reduction in scavenged OH when isoprene SOA and Fe(II) are mixed in SLF. They hypothesized that these reactive species are scavenged by ascorbate and other antioxidants, with concurrent production of the ascorbyl radical. These results indicate that OH produced from SOA and from Fe(II) + ROOH/ROOR reactions leads to efficient oxidation of AA to DHA and an increase in OP_AA_.

#### 3.5.3 NSOA + AA

The NSOA-specific model was built from an additional 16 reactions from the literature (R90–106, Table S2) and reported yields of 1,2NQN and 1,4NQN from NSOA formed from naphthalene photooxidation.^12^ The resulting model is in very good agreement with the OP_AA_ measurements, coming within about 95%. To the authors’ knowledge, this model is the first to include the reaction of AA/AH^-^ and naphthoquinones specific to NSOA, including different rate constants for quinone isomers and AA/AH^-^. Direct reactions of quinones with AA/AH^–^ dominate DHA formation; 1,2 naphthoquinone (1,2NQN) is responsible for ∼90% of DHA formation via the reactions of 1,2-NQN with AA/AH^–^, producing the ascorbyl radical (A^.–^) which promptly undergoes disproportionation to form DHA (R12, R13, R90–100, Table S2). The reaction between AA and 1,4 naphthoquinone (1,4-NQN) contributes an additional 10% to DHA formation through a mechanism analogous to 1,2-NQN.

#### 3.5.4 BSOA + AA + Fe(II)

The model is less successful in reproducing OP_AA_ measurements of Fe(II) + BSOA. The Fe(II) + BSOA model assumes Fenton-like reactions between ROOH present in BSOA and Fe(II) (R112, Table S2). However, OP_AA_ measurements (Figure 5) show that the OP_AA_ signal from Fe(II) + BSOA is less than the sum of OP_AA_ from Fe(II) and BSOA separately when Fe(II) and BSOA are mixed (Figure 5). Although the source of the discrepancy is not clear, the kinetic model does not consider complexation of Fe(II) by chelating organics present in BSOA, such as carboxylic acids and carbonyl groups, which have been shown to both enhance and suppress Fe(II) redox activity.^51,57^ In addition, (di)carboxylic acids such as pinic and pinonic acid are abundant oxidation products in BSOA.^58^ The interaction of these species with Fe(II) which is not included in the model may explain this discrepancy.

#### 3.5.5 NSOA + AA + Fe(II)/Cu(II)

The model is in reasonably good agreement with OP_AA_ measurements for Fe(II) + NSOA, slightly underpredicting OP_AA_. NSOA formed via photooxidation has been shown to contain large quantities of HULIS-like molecules, with yields reported up to 30%.^50^ HULIS has been shown to complex Fe(II), enhancing the rate of redox reactions.^14^ The model includes an estimate of Fe(II) complexation by HULIS-like molecules derived from experiments using Suwannee River Fulvic Acid (SRFA) as a surrogate for HULIS, as described in Gonzalez et al.^14^ The enhanced Fenton chemistry associated with Fe(II)-HULIS + H_2_O_2_ (R123 Table S2) increases the contribution of OH to DHA formation to 22% compared to 11% for Fe(II) only. This mechanism broadly describes the synergistic enhancement of the measured OP_AA_ of Fe(II) + NSOA, highlighting the potentially important role of metal–organic complexation with regard to increased OP_AA_. In contrast to Fe(II) + NSOA, for Cu(II) + NSOA the model underpredicts DHA formation and does not capture the synergy observed in the measurements, instead predicting a value that is essentially equal to the sum of Cu(II) and NSOA measured separately. The Cu(II) + NSOA model does not contain any HULIS-Cu(II) complexation, which may influence Cu(II) redox chemistry in a manner analogous to Fe(II)-HULIS. Tong et al.^59^ observed that radical production from Cu(II) + cumene hydroperoxide increased in the presence of humic acid, and at higher concentrations of humic acid, the yield of OH increased.^59^

## 4 Atmospheric Implications

The oxidative potential (OP) of particulate matter has been widely suggested as a key metric for describing particle toxicity. The emergence of acellular OP assays has led to a rapid increase in research interest and application of OP measurements globally. In some cases, OP measurements outperform the policy standard of PM_2.5_ mass concentrations regarding prediction of health outcomes.^3^ However, large uncertainty remains regarding the relationship between particle chemical composition, including particle-phase interactions of chemical species and aqueous-phase chemistry occurring in, e.g., the lung, and OP. Developing our understanding of the relationship between aerosol chemical composition, often with unique emission sources, and OP is crucial in order to develop more source-specific air pollution mitigation strategies. In particular, understanding the chemical interactions of key components, such as SOA and redox-active transition metals, and their influence on OP is crucial. This is particularly important as contributions of nonexhaust emissions, dominant sources of Cu and Fe in an urban environment, are predicted to steadily grow in the future due to increase in electric car use, stringent policies regarding tailpipe emissions (i.e., lowering tailpipe emissions), and lack of policies focused on nonexhaust emissions.^60^

This study presents the first simultaneous application of two online methods to quantify OP_AA_ and ROS_DCFH_ in a laboratory setting, providing robust and accurate quantification of the oxidative properties of biogenic and anthropogenic SOA. The simultaneous application of online instruments capture rapid chemistry that traditional filter-based method may not fully characterize, particularly the reaction of labile and reactive peroxides, which our previous study shows decrease by up to 90% prior to offline analysis.^27^ Therefore, the use of online methods allows the quantification of highly reactive peroxides, and their reactions with Fe(II) and Cu(II), providing key new insights into the role this chemistry plays in particle OP. All assays show that NSOA, a surrogate for anthropogenic SOA, has intrinsically higher ROS_DCFH_, OP_AA_, and OP_OH_, in agreement with our previous studies.^27,61^ ROS_DCFH_ measurements indicate the enhanced destruction of organic peroxides by redox-active Fe(II) and Cu(II) chemistry, leading to a decrease in ROS_DCFH_ in both BSOA and NSOA. Complementary online OP_AA_ and filter-based OP_OH_ measurements show synergistic enhancements of OP_AA_ when SOA is mixed with Fe(II) and Cu(II). Interestingly, OP_AA_ and OP_OH_ are particularly enhanced when Cu(II) is mixed with BSOA. A decrease in ROS_DCFH_, which predominantly measures organic peroxides, would suggest that decomposition of peroxides by Cu(II) liberates more reactive species such as O_2_^•–^ and OH, which oxidize AH^–^ faster than peroxides, therefore leading to an increase in OP_AA_ and OP_OH_.

Our kinetic model provides additional insight into the mechanisms that lead to observed OP_AA_ for SOA, Fe(II), Cu(II), and metal–SOA mixtures, where in general the model is in good agreement with OP_AA_ measurements. Model results suggest that the direct reactions of Fe(II)/Fe(III) and Cu(II) as well as 1,2-NQN with AH^–^ are key contributors to OP_AA_. Fe(II)–HULIS reactions may be at least partially responsible for the observed enhancement of OP_AA_ and OP_OH_ when Fe(II) and NSOA are mixed. The key results of this study demonstrate that the interaction of Fe(II) and Cu(II) with NSOA and BSOA results in a range of synergistic and antagonistic enhancements.

Furthering our understanding of key chemical mechanisms that influence OP will provide vital information regarding the influence of chemical composition on OP and hence health relevant properties of particles, helping to build toward more targeted and efficient air pollution mitigation strategies.

## Supporting Information Available

Additional experimental details, methods and materials used, SMPS data for SOA and metal particles produced, representative online OP data, ROS_DCFH_ and OP_AA_ responses to a range of commercially available compounds, MINTEQ modeling data, OH production from Cu(II) and H_2_O_2_, and the reactions used for kinetic modeling (PDF)
