# Supplementary material for: Iron and Copper Alter the Oxidative Potential of Secondary Organic Aerosol: Insights from Online Measurements and Model Development
Source: Environ Sci Technol. 2023 Aug 25;57(36):13546–58. doi: 10.1021/acs.est.3c01975 (PMC10501117; doi:10.1021/acs.est.3c01975)
Supplement: Supplementary file 1 — es3c01975_si_001.pdf [file es3c01975_si_001.pdf]

Supplementary Information for

Iron and Copper Alter the Oxidative Potential of Secondary Organic Aerosol: Insights from Online Measurements, and Model Development

Steven J. Campbell[§,‡,*], Battist Utinger[§], Alexandre Barth[§], Suzanne E. Paulson[‡], and Markus Kalberer[§]

[§]Department of Environmental Sciences, University of Basel, Klingelbergstrasse 27, 4057, Basel, CH

[‡]Department of Atmospheric and Oceanic Sciences, University of California at Los Angeles, 520 Portola Plaza, Los Angeles, CA.

This PDF file includes: 21 total pages, 8 Figures, 2 Tables.

Table of Contents

S1. Materials and Methods *3*

S1.1 Reagents 3

S1.2 Metal Particle and Secondary Organic Aerosol Generation using the Organic Coating Unit (OCU) 3

S1.3 Online Particle bound ROS instrument (OPROSI, OP_{DCH}) 5

S1.4 Online Oxidative Potential Ascorbic Acid Instrument (OOPAAI, OP_{AA}) 6

S1.5 Organic Coating Unit (OCU) Experimental Conditions 7

S2. Results *8*

S2.1 Representative Online Data. 8

S2.2 OP_{DCFH} and OP_{AA} Responses to a Range of Peroxides, Metals and Quinones 10

S2.3 MINTEQ Modelling 12

S2.4 OH production from Cu (II) and H_2O_2 13

S2.5 Kinetic Modelling 15

S1. Materials and Methods

S1.1 Reagents

Reagents were all purchased from Merck unless otherwise stated. *L*-ascorbic acid (≥ 99%), *L*-Dehydroascorbic acid, H_2O_2 solution (3%), Chelex 100 sodium form, 1 M HCl solution, 0.1 M NaOH solution, 1 M PBS solution, HEPES (≥ 99.5%), $CuSO_4$ (≥ 99%), $FeSO_4$ (≥ 99%), $Fe_2(SO_4)_3$ (≥ 98%), *o*-phenylenediamine (≥ 99.5%), β-pinene (98%), Naphthalene (98%), horseradish peroxidase (Type VI) and 2,7-dichlorofluoroscein diacetate (98%). Disodium Terepthalate and 2-hydroxyterepthalic acid for OP_{OH} measurements were purchased from TCI (USA). All aqueous solutions were prepared using water obtained from a Merck Synergy high-purity water unit (resistivity ≥ 18.2 MΩ cm^{-1}). High-purity water was further purified by flowing through a 10 cm column packed with Chelex-resin at a flow rate of 1 drop per minute, to minimise background contributions from transition metals.[28]

S1.2 Metal Particle and Secondary Organic Aerosol Generation using the Organic Coating Unit (OCU)

A flow through system consisting of various components to facilitate the production of secondary organic aerosol, metal seed particles and mixed metal-organic particles prior to analysis with the OPROSI and OPOAAI is displayed in Figure S1. Fe (II) and Cu (II) particles were produced using a homebuilt nebuliser containing a 0.4 mM solution of $FeSO_4$ or $CuSO_4$. The resulting particles were dried with a silica denuder, and then passed through an organic coating unit (OCU) (see Keller et al.).[1] In short, the OCU maintains a steady concentration of the gas phase volatile organic compound (VOC) precursor, which is maintained via a PID voltage feedback. For BSOA production, 1 mL of β-pinene was placed in a VOC reservoir at room temperature with a steady gas flow passed over the surface. For NSOA production, 1 g of naphthalene was placed in the VOC reservoir and heated to 80 ^{o}C using a water bath to volatilise

the naphthalene into the gas phase. The VOCs were then passed through a cylindrical quartz photooxidation chamber (76 ml volume) surrounded by 5 low pressure mercury lamps (4W UVC with 254 nm and 185 nm emission lines, type GPH212T5VH/2, Heraeus, Germany), which produce both O_3 and OH radicals via the photolysis of O_2 under humid conditions. Only one UV lamp was turned on for all online photooxidation experiments presented in this study resulting in a maximum O_3 concentration of 2 x 10^{13} molecules cm^{-3} and an estimated OH concentration in the chamber of ~1 x 10^{9} molecules cm^{-3} in the absence of VOCs, with a constant gas flow of 1 L/min through the oxidation chamber. We expect predominantly OH-initiated β-pinene SOA due to the greater rate constant associated with OH reaction (7.9 x 10^{-11} molecules cm^{-3} s^{-1}) at the exocyclic double bond compared to O_3 (1.5 x 10^{-17} molecules cm^{-3} s^{-1}), whereas naphthalene oxidation proceeds only through OH initiated oxidation.[2] All experiments were performed at 70 ± 20 % RH in the chamber, which was maintained using a humidifier in the OCU. The resulting SOA mixture is then passed through a series of charcoal denuders to remove gas phase VOCs (Figure S1) and remove ~99.9% O_3.[3] Gas phase VOC concentrations were monitored online using a proton transfer time-of-flight mass spectrometer (PTR-ToF-MS, 3000, Ionicon, Innsbruck, Austria). Particle number size distributions, were constantly monitored using a scanning mobility particle sizer (SMPS, TSI). A particle density of 1.3 g cm^{-3} was assumed to calculate particle mass distributions. O_3 concentrations were continuously monitored using an O_3 analyser (Model 49i, Thermo Scientific). The particle-bound ROS concentrations and particle oxidative potential were then measured online using the OPROSI and OOPAAI, respectively, the operational procedures of which are described below. SOA particles were generated in the absence of NO_x. This results in a more dominant formation of hydroperoxides and can be considered as representative of conditions encountered in the unpolluted atmosphere.

For OH analysis, SOA particles were collected on PTFE membrane filters (Whatman, TE 35, 0.2 µm pore size) at a flow rate of 5 L min^{-1}. To reduce any background contamination, they

were soaked and washed with methanol before use. The OCU was optimised to maximise the SOA output. All 5 lamps as well as the highest amount of VOC were in use. A total mass of 920± 166 µg was collected per filter. This resulted in a collection time of 20 minutes for the BSOA and 60 minutes for the NSOA samples. The loaded filters were stored at -20°C after collection.

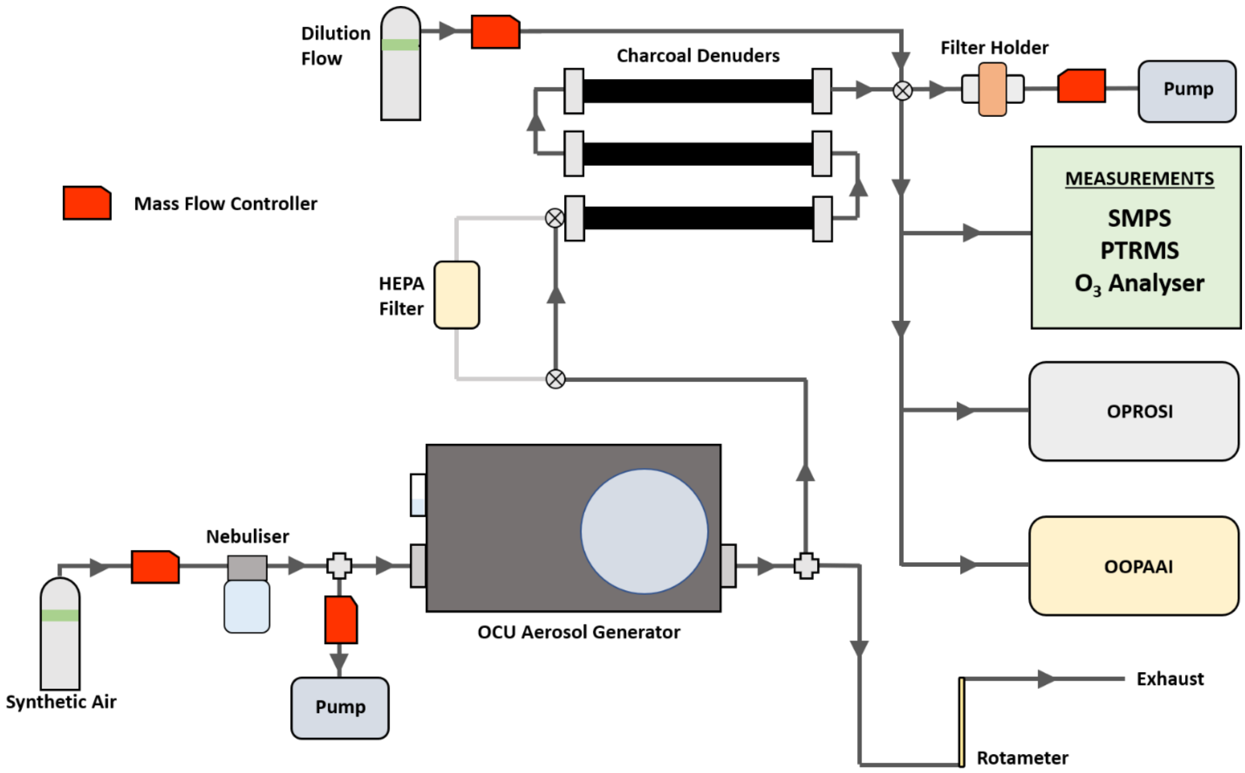

Figure S1 - Schematic representation of the experimental setup used in this study.

S1.3 Online Particle bound ROS instrument (OPROSI, OP_{DCH})

The functionality, design and operating procedure for the OPROSI is extensively described in Wragg et al.[4] and Fuller et al.[5] Briefly, the aerosol is continuously drawn into the instrument via an aerosol conditioning unit, which consists firstly of a stainless steel cyclone (2.5 µm cut-off at 5 L/min URG-2000-30E-5-2.5-S, URG) and charcoal denuder, before entering a homebuilt particle sampler. Particles are collected onto a filter sprayed with a solution of horseradish peroxidase (HRP) in 10% PBS buffer at 1 ml min^{-1}, which immediately reacts with

ROS present in the particles and is collected in a 1 mL liquid reservoir. The HRP solution is then immediately mixed with 2,7-diclhorofluoroscein (DCFH), which is subsequently oxidised to form a fluorescent product DCF by the ROS-HRP solution in a reaction bath maintained at 37 ^{o}C for 15 minutes. The DCF is then quantified via fluorescence spectroscopy. The fluorescence response of the instrument is calibrated with known concentrations of hydrogen peroxide (H_2O_2), and thus ROS concentrations are expressed in H_2O_2 equivalent concentrations per unit volume (m^{-3}) or per unit particle mass (ug^{-1}). The assay has demonstrated sensitivity in particular to hydrogen peroxides and organic peroxides.[5,4] The direct-to-liquid sampling and high time resolution of this instrument therefore is particularly sensitive to short-lived ROS components, which react within seconds after sampling.[5,4]

S1.4 Online Oxidative Potential Ascorbic Acid Instrument (OOPAAI, OP_{AA})

The initial iteration of the OOPAAI is described in detail in Campbell et al.,[6] was used, with substantial modifications, including technical and chemical improvements presented in the following description, most notably running the assay with a HEPES buffer at pH 6.8 (Utinger et al).[7] The OOPAAI measures OP_{AA} by measuring the formation of dehydroascorbic acid (DHA), an oxidation product of ascorbic acid (AA), by reacting DHA with *o*-phenylenediamine (OPDA) forming a fluorescent product 3-(1,2-dihydroxyethyl)-fluoro-[3,4-b]quinoxaline-1-one (DFQ), where we then monitor the concentration of DFQ using fluorescence spectroscopy.

Aerosol particles are continuously collected for online OP analysis using a commercially available particle-into-liquid sampler (PILS, Brechtel, USA), where the wash flow was modified to contain the AA reagent, ensuring rapid reaction of AA of aerosol particle components with direct-to-liquid particle sampling. The sample is then washed off the impactor with a flow rate of 60 µl/min and the resulting AA-particle aqueous sample is reacted for 10 minutes at 37 ^{o}C. The reaction solution is then mixed with OPDA at a flow of 90 µl/min and

pumped into another reaction coil at room temperature for 2 min, where the DHA + OPDA reaction occurs, forming the fluorescent product DFQ. The concentration of DFQ is then monitored using a homebuilt flow through fluorescence cell which consisted of a modified flow-through quartz cuvette (Hellma Analytics). DFQ is excited by a high-power UV LED (Roithner Lasertechnik, type UVLED- 365-330-SMD) at 365 nm via an optical fiber (Thorlabs, 1500 µm, NA 0.39). The fluorescence emission light is then collected through a collimating lens (Ocean Insight) via an optical fiber (Thorlabs, 1500 µm, NA 0.50) and then detected using a spectrometer (Ocean Insight, QePro). The OOPAAI is calibrated using known concentrations of DHA, and hence the OP here is then expressed in terms of nmol DHA per unit volume (m^{-3}) or unit mass (µg m^{-3}).

S1.5 Organic Coating Unit (OCU) Experimental Conditions

Particle mass concentrations used in this study are presented in Table S1. A particle mass concentration range between 245-408 µg m^{-3} for SOA produced from the OCU and 5-34 µg m-3 for metal particles. OH concentrations in the photolysis chamber were estimated to be ~1 x 10^9 cm^{-3} , based on the rate of decay of gas phase naphthalene present in the system.

Table S1 – Reaction conditions (particle mass, metal mass, O3, OH, RH,) – emphasise metal ratio

Experiment	Organic SOA (µg m^{-3})	Metal (µg m^{-3})
NAP + Fe (II)	351 ± 70.2	34 ± 6.8
NAP + Cu (II)	356 ± 71.2	5.74 ± 1.15
BP + Fe (II)	245 ± 49	36 ± 8.7
BP + Cu (II)	408 ± 81.6	5.4 ± 1.08

S2. Results

S2.1 Representative Online Data.

The representative size distributions of SOA, metal and a SOA-metal mixture are presented in Figure S2. We assume that the SOA and metals during the mixed aerosol experiments are internally mixed due to the one mode observed in the size distribution.

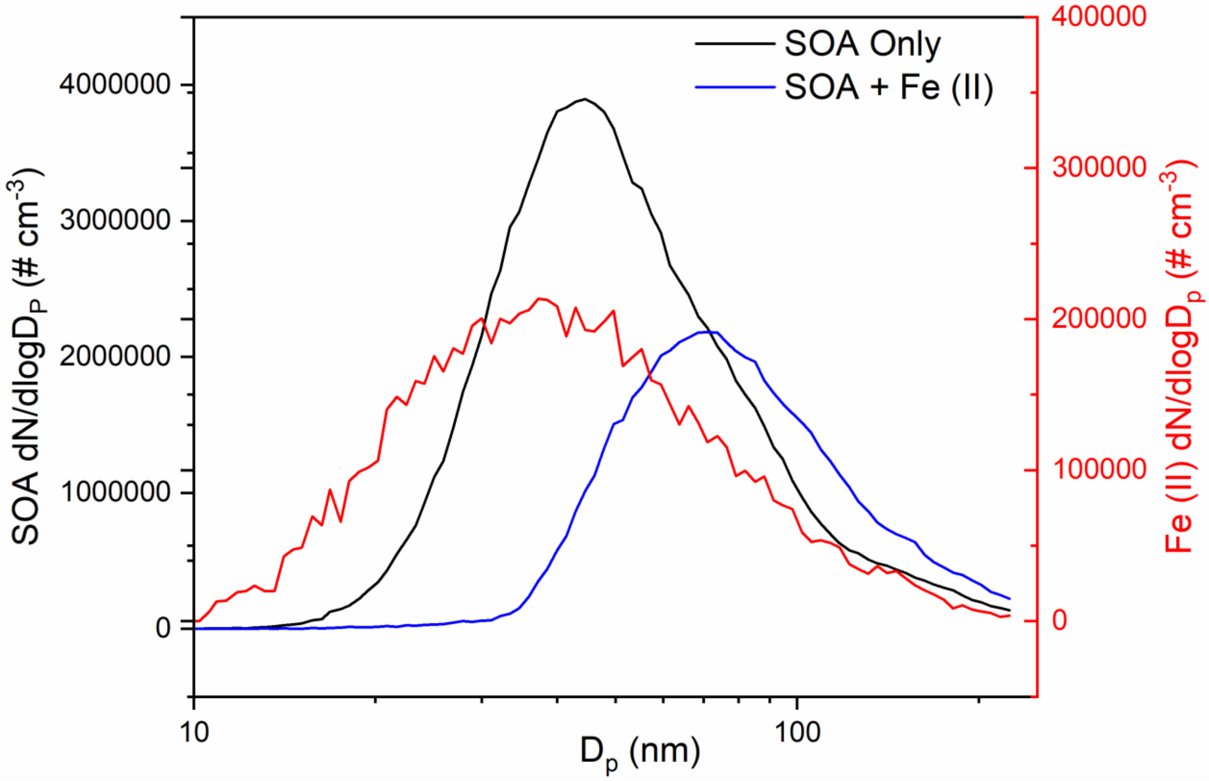

Figure S2 – Size distributions of secondary organic aerosol, nebulised Fe_2SO_4 (red, right y-axis) and a mixture of both, showing well mixed aerosol SOA/metal aerosol particles.

Using the experimental setup described in Figure S1, online particle-bound ROS and particle OP were quantified for ß-pinene-derived SOA (BSOA), naphthalene derived SOA (NSOA) and redox active transition metals including Fe (II) and Cu (II). Figure S3 shows a representative plot illustrating the online raw signal response of the OPROSI over 3.5 hours quantifying OP_{DCFH} of pure Cu (II), pure BSOA or Cu (II) + BSOA particles. Total particle mass (SMPS measurements) is also displayed. Particles are well mixed as evidenced by the growth of particle size distribution, and one mode observed for SOA + metal mixtures in the OCU (Figure S2).

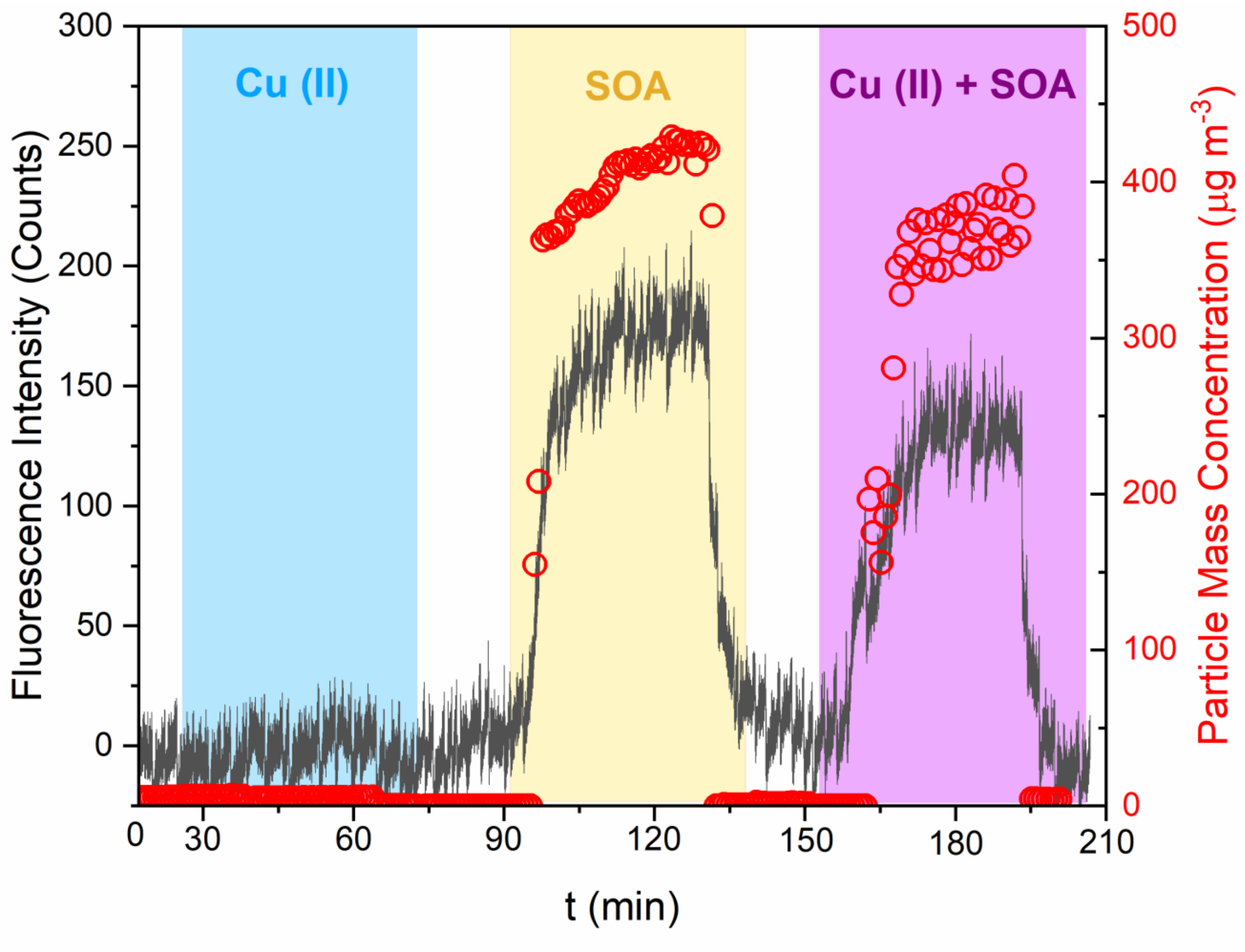

Figure S3 –Representative online data illustrating the OPROSI (measuring OP_{DCFH}) response to Cu (II) (blue), ß-pinene SOA (yellow), and a mixture of Cu (II) and ß-pinene SOA (purple) and corresponding total particle mass.

S2.2 OP_{DCFH} and OP_{AA} Responses to a Range of Peroxides, Metals and Quinones

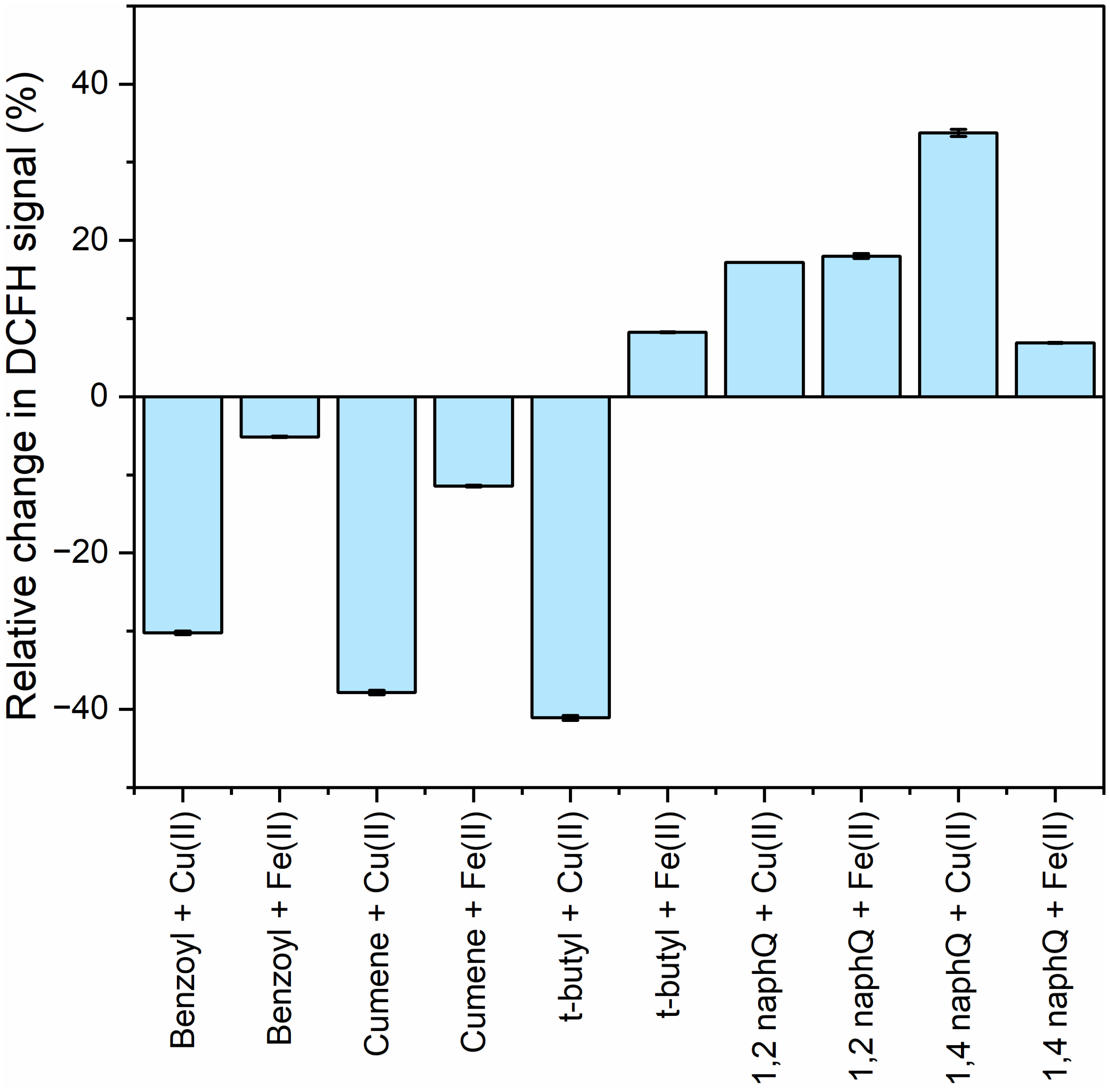

Figure S4 – OP_{DCFH} response when a range of commercially available peroxides and quinones are mixed with Fe (II) and Cu (II).

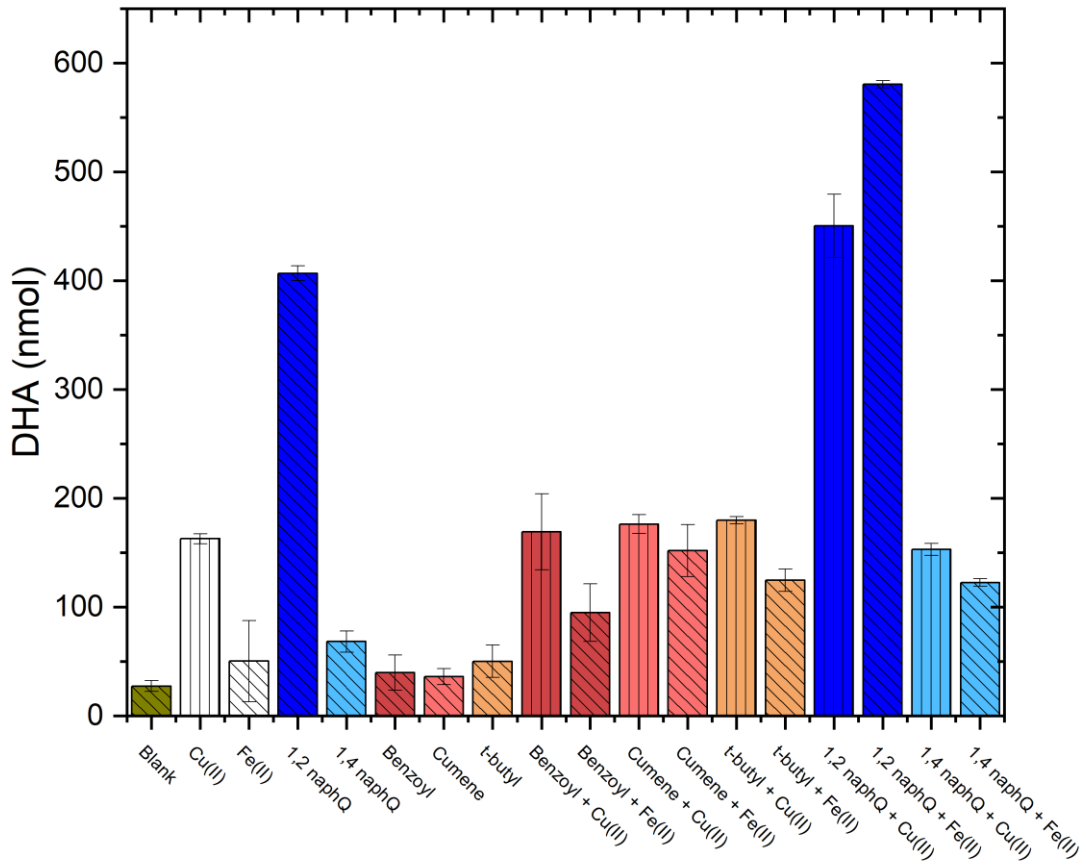

Figure S5 – DHA formation (i.e OP_{AA}) response for a range of commercially available peroxides and naphthoquinones, as well as Fe (II) and Cu (II), and mixtures of a range of peroxides and naphthoquinones. .

S2.3 MINTEQ Modelling

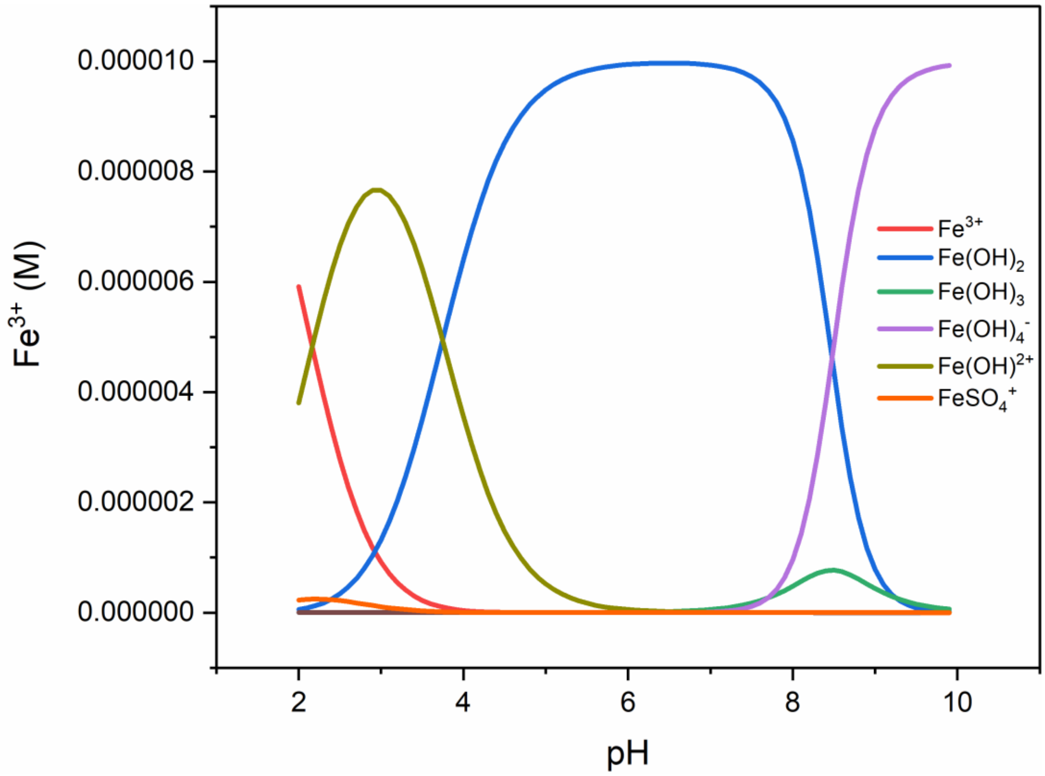

Figure S6 – MINTEQ model describing Fe (III) speciation as a function of pH.

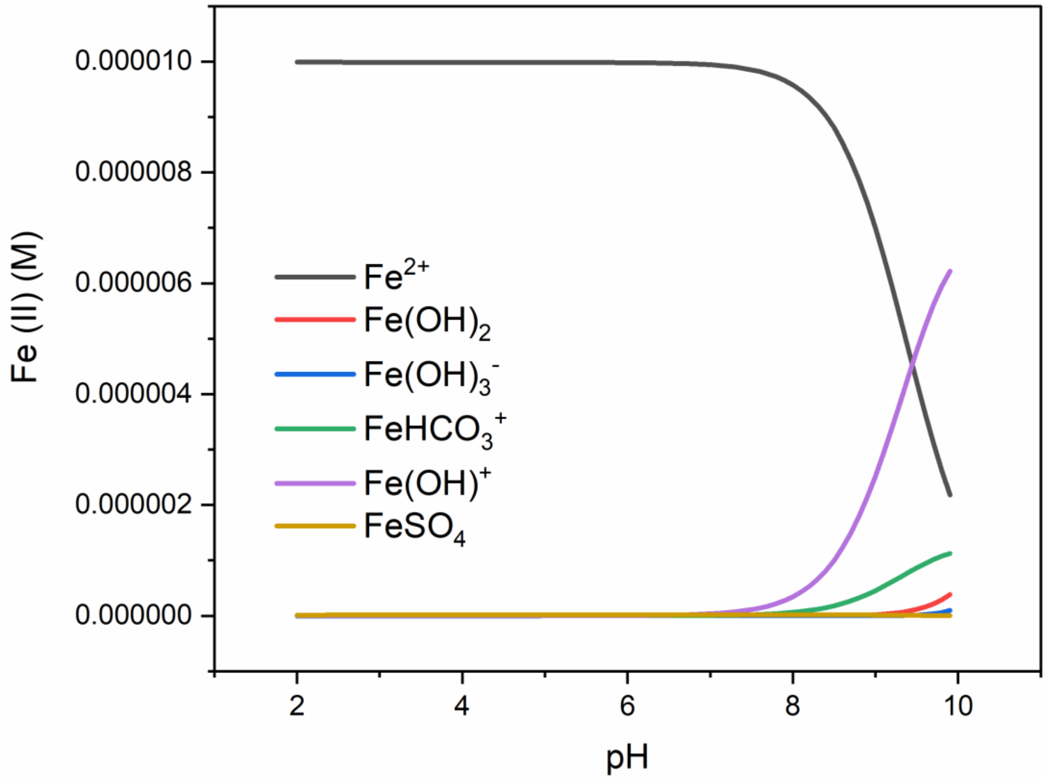

Figure S7 – MINTEQ model describing Fe (II) speciation as a function of pH.

S2.4 OH production from Cu (II) and H_2O_2

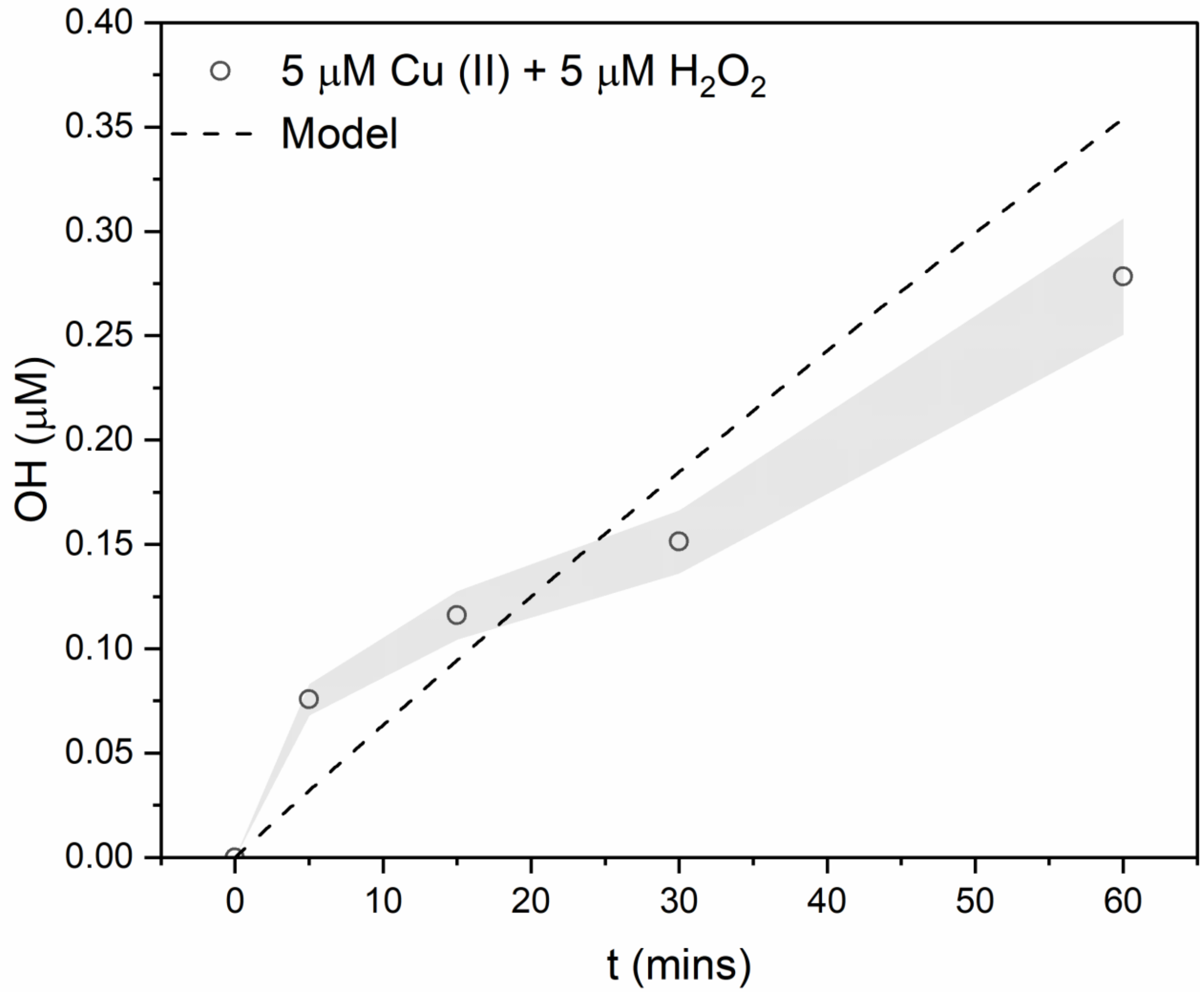

Figure S8 – Comparison between OH measurements of a mixture of 5 µM Cu (II) and 5 µM H_2O_2.

S2.5 Kinetic Modelling

Table S2 – Kinetic Model

No.	Reaction	k_f (M^{-1} s^{-1}, s^{-1})	k_b (M^{-1} s^{-1}, s^{-1})	K_{eq} (M^{-1} s^{-1}, s^{-1})	ref	Notes
	**Ascorbate Reactions**					
1	$AH_2 \rightleftharpoons AH^- + H^+$			7.94×10^{-5}	8	
2	$AH^- \rightleftharpoons A^{2-} + H^+$			1.58×10^{-12}	8	
3	$AH \rightleftharpoons A^{\cdot-} + H^+$			7.24	8	
4	$AH_2 + OH^{\cdot} \rightarrow A^{\cdot-} + H_2O + H^+$	7.9×10^{9}			9	
5	$AH_2 + HO_2^{\cdot} \rightarrow A^{\cdot-} + H_2O_2 + H^+$	1.6×10^{4}			10	
6	$AH_2 + O_2^{\cdot-} \rightarrow A^{\cdot-} + H_2O_2$	5.48×10^{7}			11	
7	$AH^- + OH^{\cdot} \rightarrow A^{\cdot-} + H_2O$	1.1×10^{10}			8	
8	$AH^- + HO_2^{\cdot} \rightarrow A^{\cdot-} + H_2O_2$	1.25×10^{6}			10	
9	$AH^- + O_2^{\cdot-} \rightarrow A^{\cdot-} + HO_2^-$	5×10^{4}			10	
10	$A^{\cdot-} + HO_2^{\cdot} \rightarrow DHA + HO_2^-$	5×10^{9}			11	
11	$A^{\cdot-} + O_2^{\cdot-} \rightarrow DHA + HO_2^- - H^+$	2.6 $x10^{8}$			11	
12	$2A^{\cdot-} \rightleftharpoons AH^- + DHA - H^+$	5×10^{4}	2×10^{-12}	1.67×10^{16}	12	
13	$2A^{\cdot-} \rightleftharpoons AH_2 + DHA - 2H^+$	8×10^{7}	3.8×10^{-13}	2.1×10^{20}	12	
14	$Fe(III)^{**} + AH_2 + O_2 \rightarrow Fe(III)^{**} + DHA + H_2O_2$	5.7×10^{4}			13	
15	$Fe(III)^{**} + AH^- + O_2 \rightarrow Fe(III)^{**} + DHA + H_2O_2 - H^+$	4.7×10^{4}			13	
16	$Cu(II)^{**} + AH_2 + O_2 \rightarrow Cu(II)^{**} + DHA + H_2O_2$	1×10^{5}			13	
17	$Cu(II)^{**} + AH^- + O_2 \rightarrow Cu(II)^{**} + DHA + H_2O_2 - H^+$	2.5×10^{6}			13	
	**DHA Reactions**					
18	$oPDA + DHA \rightarrow DHA - oPDA$	4.6			14	
19	$DHA + OH^{\cdot} \rightarrow product$	1×10^{10}				Assumed to be the same as AA + OH
20	$DHA + H_2O \rightarrow DKG$	5.8×10^{-4}			15	
21	$DKG + OH^{\cdot} \rightarrow product$	1×10^{10}				Assumed to be the same as AA + OH
22	$DHA + H_2O_2 \rightarrow products$	3.4×10^{-2}			16	
	**Fenton Reactions**					
23	$H_2O_2 + Fe(II) \rightarrow {}^{-}OH + \bullet OH$	63-76			17	
24	$H_2O_2 + Fe(II) \rightarrow Fe(IV)O^{2+} + H_2O$	63-76			18	
25	$H_2O_2 + Fe(III) \rightarrow HO_2\bullet + Fe(II) + H^+$	0.01-0.001			19,20	
26	$H_2O_2 + \bullet OH \rightarrow HO_2\bullet + H_2O$	3.3×10^{7}			17,21,22	
27	$H_2O_2 + 2\ Fe(IV)O_2^{+} \rightarrow HO_2\bullet + Fe^{3+} + {}^{-}OH$	1×10^{4}			23	
	**ROS Reactions**					
28	$OH^{\cdot} + OH^{\cdot} \rightarrow H_2O_2$	5.5×10^{9}			24	
29	$H_2O_2 + OH^{\cdot} \rightarrow HO_2^{\cdot} + H_2O$	3.2×10^{7}			25	
30	$O_2^{\cdot-} + OH^{\cdot} \rightarrow OH^- + O_2$	1.01×10^{10}			24	
31	$HO_2^{\cdot} + OH^{\cdot} \rightarrow H_2O + O_2$	7.1×10^{9}			24	
32	$O_2^{\cdot-} + H_2O_2 \rightarrow OH^- + OH^{\cdot} + O_2$	0.13			24	
33	$O_2^{\cdot-} + O_2^{\cdot-} \rightarrow O_2 + H_2O_2 - 2H^+$	6.0×10^{5}			26	
34	$HO_2^{\cdot} + O_2^{\cdot-} \rightarrow HO_2^- + O_2$	9.7×10^{7}			24	
35	$H_2O_2 + HO_2^{\cdot} \rightarrow H_2O + O_2 + OH^{\cdot}$	0.5			27	
36	$HO_2^{\cdot} + HO_2^{\cdot} \rightarrow O_2 + H_2O_2$	8.3×10^{5}			24	

37	$O_2^{2-} + H^+ \rightarrow HO_2^-$	1×10^{10}			[24]	
38	$HSO_4^- + OH^\cdot \rightarrow SO_4^- + H_2O$	3.5×10^{5}			[28]	
	**General Equillibria**					
39	$H_2O \rightleftharpoons H^+ + OH^-$	1.3×10^{-3}	1.3×10^{11}	1×10^{-14}	[27]	
40	$H_2O_2 \rightleftharpoons H^+ + HO_2^-$	1.26×10^{-2}	1×10^{10}	1.26×10^{-12}	[27]	
41	$HO_2 \rightleftharpoons H^+ + O_2^{\cdot -}$	1.14×10^{6}	7.2×10^{10}	1.58×10^{-5}	[25]	
42	$H^+ + SO_4^{2-} \rightleftharpoons HSO_4^-$			97.7	[27]	
	**Inorganic Fe (II)/Fe (III) Reactions**					
43	$Fe^{3+} + H_2O \rightleftharpoons FeOH^{2+} + H^+$			6.11×10^{-3}	[28]	
44	$FeOH^{2+} + H_2O \rightleftharpoons Fe(OH)_2^+ + H^+$			7.78×10^{-6}	[28]	
45	$Fe^{2+} + H_2O \rightleftharpoons FeOH^+ + H^+$			3.16×10^{-10}	[27]	
46	$Fe^{3+} + SO_4^{2-} \rightleftharpoons FeSO_4^+$			8.32×10^{3}	[27]	
47	$Fe^{3+} + 2SO_4^{2-} \rightleftharpoons Fe(SO_4)_2^-$			2.63×10^{5}	[27]	
48	$Fe^{2+} + SO_4^{2-} \rightleftharpoons FeSO_4$			1.78×10^{2}	[27]	
49	$Cl^- + Fe^{3+} \rightleftharpoons FeCl^{2+}$	3×10^{3}	2.16×10^{3}	1.39	[27]	
50	$Fe^{2+} + O_2 \rightarrow Fe^{3+} + O_2^{\cdot -}$	3.9			[26]	
51	$Fe(III)^* + O_2^{\cdot -} \rightarrow Fe^{2+} + O_2$	5×10^{7}			[27]	
52	$FeSO_4^+ + O_2^{\cdot -} \rightarrow Fe^{2+} + SO_4^{2-} + O_2$	$<1\times10^{3\Delta}$			[27]	
53	$Fe(SO_4)_2^- + O_2^{\cdot -} \rightarrow Fe^{2+} + 2SO_4^{2-} + O_2$	$<1\times10^{3\Delta}$			[27]	
54	$Fe(III)^* + HO_2^\cdot =\rightarrow +O_2 + H^+$	2×10^{4}			[27]	
55	$FeSO_4^+ + HO_2^\cdot \rightarrow Fe^{2+} + SO_4^{2-} + O_2 + H^+$	$<1\times10^{3\Delta}$			[27]	
56	$Fe(SO_4)_2^- + HO_2^\cdot \rightarrow Fe^{2+} + 2SO_4^{2-} + O_2 + H^+$	$<1\times10^{3\Delta}$			[27]	
57	$Fe^{3+} + H_2O_2 \rightarrow Fe(HO_2)^{2+} + H^+$	3.1×10^{7}	1×10^{10}	3.1×10^{-3}	[27]	
58	$FeOH^{2+} + H_2O_2 \rightarrow Fe(OH)(HO_2)^+ + H^+$	2×10^{6}	1×10^{10}	2×10^{-4}	[27]	
59	$Fe(II)^* + OH^\cdot \rightarrow Fe^{3+} + OH^-$	2.7×10^{8}			[27]	
60	$FeSO_4 + OH^\cdot \rightarrow Fe^{3+} + SO_4^{2-} + OH^-$	2.7×10^{8}			[27]	
61	$Fe(II)^* + O_2^{\cdot -} \rightarrow +O_2^{2-}$	1×10^{7}			[27]	
62	$FeSO_4 + O_2^{\cdot -} \rightarrow Fe^{3+} + SO_4^{2-} + O_2^{2-}$	5×10^{8}			[27]	
63	$Fe(II)^* + HO_2^\cdot \rightarrow Fe^{3+} + HO_2^-$	1.2×10^{6}			[27]	
64	$FeSO_4 + HO_2^\cdot \rightarrow Fe^{3+} + SO_4^{2-} + HO_2^-$	1.2×10^{6}			[27]	
65	$Fe^{2+} + H_2O_2 \rightarrow Fe^{3+} + OH^\cdot + OH^-$	55			[27]	
66	$FeOH^+ + H_2O_2 \rightarrow Fe^{3+} + OH^\cdot + 2OH^-$	55			[27]	
67	$FeSO_4 + H_2O_2 \rightarrow Fe^{3+} + SO_4^{2-} + OH^\cdot + OH^-$	78			[27]	
68	$Fe(HO_2)^{2+} \rightarrow HO_2^\cdot + Fe^{2+}$	2.3×10^{-3}			[27]	
69	$Fe(OH)(HO_2)^+ \rightarrow Fe^{2+} + HO_2^\cdot + OH^-$	2.3×10^{-3}			[27]	
	**Inorganic Cu(I)/Cu(II) Reactions**					
70	$Cu^{2+} + H_2O \rightleftharpoons CuOH^+ + H^+$			1.12×10^{-8}	[29]	
71	$Cu^{2+} + 2H_2O \rightleftharpoons Cu(OH)_2 + 2H^+$			6.31×10^{-17}	[29]	
72	$Cu^{2+} + 3H_2O \rightleftharpoons Cu(OH)_3^- + 3H^+$			2.51×10^{-27}	[29]	
73	$Cu^{2+} + 4H_2O \rightleftharpoons Cu(OH)_4^{2-} + 4H^+$			1.82×10^{-40}	[29]	
74	$2Cu^{2+} + H_2O \rightleftharpoons Cu_2OH^{3+} + H^+$			3.98×10^{-7}	[29]	
75	$2Cu^{2+} + 2H_2O \rightleftharpoons Cu_2(OH)_2^{2+} + 2H^+$			3.72×10^{-11}	[29]	

76	$3Cu^{2+} + 4H_2O \rightleftharpoons Cu_3(OH)_4{}^{2+} + 4H^+$			7.94×10^{-22}	[29]	
77	$Cu^{2+} + SO_4{}^{2-} \rightleftharpoons CuSO_4$			223.9	[29]	
78	$Cu^{2+} + Cl^- \rightleftharpoons CuCl^+$			6.76	[29]	
79	$Cu^{2+} + 2Cl^- \rightleftharpoons CuCl_2$			3.98	[30]	
80	$Cu(II)^* + OH^{\cdot} \rightleftharpoons CuOH^{2+}$	1.17×10^4	3×10^4		[30]	
81	$Cu(II)^* + HO_2{}^{\cdot} \rightarrow Cu^+ + O_2 + H^+$	1×10^8			[31,32]	
82	$Cu(II)^* + H_2O_2 \rightarrow Cu^+ + O_2{}^{\cdot-} + 2H^+$	400			[33]	
83	$Cu^+ + O_2 \rightleftharpoons Cu^{2+} + O_2{}^{\cdot-}$	4.6×10^5	8×10^9		[30]	
84	$Cu^+ + OH^{\cdot} \rightarrow Cu^{2+} + OH^-$	3×10^9			[30]	
85	$Cu^+ + H_2O_2 \rightarrow Cu^{2+} + OH^{\cdot} + OH^-$	4.7×10^3			[33]	
86	$Cu^+ + H_2O_2 \rightarrow Cu^{3+} + 2OH^-$	61			[34]	
87	$Cu^+ + Cu^{3+} \rightarrow 2Cu^{2+}$	3.5×10^9			[34]	
88	$Cu^+ + HO_2{}^{\cdot} \rightarrow Cu^{2+} + H_2O_2 - H^+$	2.3×10^9			[30]	
89	$Cu^+ + O_2{}^{\cdot-} \rightarrow +H_2O_2 - 2H^+$	1×10^{10}			[30]	
**Naphthoquinone Chemistry**						
90	$AH_2 + 12NQN \rightarrow A^{\cdot-} + 12NQN^{\cdot-}$	90.3			[35]	
91	$12NQN^{\cdot-} + O_2 \rightarrow + O_2{}^{\cdot-} + 12NQN$	2.77×10^8			[36]	
92	$12NQN^{\cdot-} + O_2{}^{\cdot-} + 2H^+ \rightarrow 12NQN + H_2O_2$	1.99×10^9			[36]	
93	$AH_2 + 14NQN \rightarrow A^{\cdot-} + 14NQN^{\cdot-}$	37.9			[35]	
94	$12NQN^{\cdot-} + 12NQN^{\cdot-} \rightarrow + 12NQN + 12OHNQN$	1×10^5			[37]	Assumed to be the same as 97
95	$14NQN^{\cdot-} + O_2{}^{\cdot-} + 2H^+ \rightarrow 14NQN + H_2O_2$	1.99×10^9			[36]	
96	$14NQN + 14OHNQN \rightarrow 14NQN^{\cdot-} + 14NQN^{\cdot-}$	1700			[37]	
97	$14NQN^{\cdot-} + 14NQN^{\cdot-} \rightarrow 14NQN + 14OHNQN$	1×10^5			[37]	
98	$14NQN + AH^- \rightarrow A^{\cdot-} + 14NQN^{\cdot-} + H^+$	45			[38]	
99	$12NQN + AH^- \rightarrow A^{\cdot-} + 12NQN^{\cdot-} + H^+$	5500			[38]	
100	$14NQN^{\cdot-} + O_2 \rightarrow 14NQN + + O_2{}^{\cdot-}$	1.1×10^8	2×10^8		[39]	
101	$14OHNQN + + O_2{}^{\cdot-} \rightarrow 14NQN^{\cdot-} + H_2O_2$	8×10^4			[39]	
102	$14NQN^{\cdot-} + + O_2{}^{\cdot-} \rightarrow 14NQN + H_2O_2$	2×10^9			[39]	
103	$Fe(III) + 14OHNQN \rightarrow Fe(II) + 14NQN^{\cdot-}$	6×10^2			[39]	
104	$Fe(III) + 14NQN^{\cdot-} \rightarrow Fe(II) + 14NQN$	5×10^4			[39]	
105	$Cu(II) + 14OHNQN \rightarrow Cu(I) + 14NQN^{\cdot-}$	4×10^4			[39]	
106	$Cu(II) + 14ONQN \rightarrow Cu(I) + 14NQN^{\cdot-}$	1.2×10^7			[39]	
107	$12NQN + OH \rightarrow Prod$	1.2×10^{10}			[39]	
108	$14NQN + OH \rightarrow Prod$	1.2×10^{10}			[39]	
109	$14NQN + SO_4{}^- \rightarrow Prod$	1.2×10^8			[39]	
**Organic Peroxide Reactions**						
110	$ROOH \rightarrow RO + OH^{\cdot}$	0.0015			[40]	
111	$OH^{\cdot} + ROOH \rightarrow ROOH + HO_2$	3.3×10^5			[40]	

112	RO → R	5×10^{5}			41	
113	R + O_2 → → RO	4.8×10^{9}			41	
114	AH^- + RO → $A^{\cdot-}$ + ROH	1×10^{4}			40	
115	Fe (II) + ROOH → RO + OH^-	4×10^{4}			42	
116	Fe (II) + ROOH → RO^- + OH	4.4×10^{3}			42	
117	RH + OH → R + H_2O	1×10^{8}			42	
**Fe-HULIS Reactions**						
118	Fepp + HULIS → Fepp-HULIS	10×10^{10}	2.9×10^{4}	3.5×10^{10}	25	HULIS broad term, see Gonzalez et al. [43]
119	Feppp + HULIS → Feppp-HULIS	1.3×10^{6}			25	
120	Fepp + HULIS → FeppHULIS$_1$	6.3×10^{-3}			25	
121	Fepp-HULIS + O_2 → + $O_2^{\cdot-}$ + Feppp-HULIS	5.1			24	
122	Fepp-HULIS + + $O_2^{\cdot-}$ + 2Hp → Feppp-HULIS + H2O2	2×10^{7}			25	
123	Fepp-HULIS + H_2O_2 → Feppp-HULIS + OH + OH^-	4.3×10^{3}			24	
124	Fepp-HULIS + OH → Feppp-HULIS + OH^-	10×10^{10}			25	
125	FeppHulis1 → Feppp-HULIS	3×10^{-3}			24	
126	Feppp-HULIS + $O_2^{\cdot-}$ →FeppHULIS + O_2	2.8×10^{5}			25	
127	FepppHULIS +AH^- → FepppHULIS + Hp + $A^{\cdot-}$	10×10^{2}			8	
128	HULIS + OH → HULISox + + $O_2^{\cdot-}$	5×10^{9}			25	
129	HULIS + + $O_2^{\cdot-}$ → HULISox + H_2O_2	9×10^{3}			25	
**Terepthalate Probe**						
130	TA + OH → (Y) TAOH + (1-Y) X	4.4×10^{9}			44	
131	X + OH → z	1×10^{9}			24	Assumed to be the same as 133
132	TAOH + OH → $TAOH_{OX}$	6.3×10^{9}			44	
**Buffer Chemistry**						
133	$HEPES \rightleftharpoons H^+ + HEPES^-$			1×10^{-3}	45	
134	$HEPES^- \rightleftharpoons H^+ + HEPES^{2-}$			2.73×10^{-8}	45	
135	$H_3PO_4 \rightleftharpoons H^+ + H_2PO_4^-$			7.08×10^{-3}	46	
136	$H_2PO_4^- \rightleftharpoons H^+ + HPO_4^{2-}$			6.31×10^{-8}	46	
137	$HPO_4^{2-} \rightleftharpoons H^+ + PO_4^{3-}$			4.79×10^{-13}	46	
138	$H_2PO_4^- + OH^{\cdot} \rightarrow H_2PO_4^{\cdot} + OH^-$	2×10^{4}			47	
139	$HPO_4^{2-} + OH^{\cdot} \rightarrow HPO_4^{\cdot-} + OH^-$	1.5×10^{5}			47	
140	$PO_4^{3-} + OH^{\cdot} \rightarrow PO_4^{\cdot 2-} + OH^-$	7×10^{6}			48	

References

(1) Keller, A.; Kalbermatter, D. M.; Wolfer, K.; Specht, P.; Steigmeier, P.; Resch, J.; Kalberer, M.; Hammer, T.; Vasilatou, K. The Organic Coating Unit, an All-in-One System for Reproducible Generation of Secondary Organic Matter Aerosol. *Aerosol Sci. Technol.* **2022**, *56* (10), 947–958. https://doi.org/10.1080/02786826.2022.2110448.

(2) Seinfeld, J. H.; Pandis, S. N. *Atmospheric Chemistry and Physics: From Air Pollution to Climate Change SECOND EDITION*; 2006.

(3) Campbell, S.; Stevanovic, S.; Miljevic, B.; Bottle, S. E.; Ristovski, Z. D.; Kalberer, M. Quantification of Particle-Bound Organic Radicals in Secondary Organic Aerosol. *Environ. Sci. Technol.* **2019**, *53*, 6729–6737. https://doi.org/10.1021/acs.est.9b00825.

(4) Wragg, F. P. H.; Fuller, S. J.; Freshwater, R.; Green, D. C.; Kelly, F. J.; Kalberer, M. An Automated Online Instrument to Quantify Aerosol-Bound Reactive Oxygen Species (ROS) for Ambient Measurement and Health-Relevant Aerosol Studies. *Atmos. Meas. Tech.* **2016**, *9* (10), 4891–4900. https://doi.org/10.5194/amt-9-4891-2016.

(5) Fuller, S. J.; Wragg, F. P. H.; Nutter, J.; Kalberer, M. Comparison of On-Line and off-Line Methods to Quantify Reactive Oxygen Species (ROS) in Atmospheric Aerosols. *Atmos. Environ.* **2014**, *92*, 97–103. https://doi.org/10.1016/j.atmosenv.2014.04.006.

(6) Campbell, S. J.; Utinger, B.; Lienhard, D. M.; Paulson, S. E.; Shen, J.; Griffiths, P. T.; Stell, A. C.; Kalberer, M. Development of a Physiologically Relevant Online Chemical Assay to Quantify Aerosol Oxidative Potential. *Anal. Chem.* **2019**, *91* (20), 13088–13095. https://doi.org/10.1021/acs.analchem.9b03282.

(7) Utinger, B.; Campbell, S. J.; Bukowiecki, N.; Barth, A.; Freshwater, R.; Ruegg, H.; Kalberer, M. An Automated Online Field Instrument to Quantify the Oxidative Potential of Aerosol Particles via Ascorbic Acid Oxidation. *Atmos. Meas. Tech. Discuss.* **2023**, No. February, 1–20.

(8) Buettner, G. R.; Schafer, F. Q. Ascorbate (Vitamin C), Its Antioxidant Chemistry. *Free Radic. Biol. Med.* **2006**, No. Vitamin C, 319–335.

(9) Redpath, J. L.; Willson, R. L. Reducing Compounds in Radioprotection and Radio-Sensitization: Model Experiments Using Ascorbic Acid. *Int. J. Radiat. Biol.* **1973**, *23* (1), 51–65. https://doi.org/10.1080/09553007314550051.

(10) Nadezhdin, A. D.; Dunford, H. B. The Oxidation of Ascorbic Acid and Hydroquinone by Perhydroxyl Radicals. A Flash Photolysis Study. *Can. J. Chem.* **1979**, *57* (12), 3017–3022.

(11) Cabelli, D. E.; Comstock, D. A.; Bielski, B. H. J. Free Radical Mechanisms for the Oxidation of Substituted Ascorbates. A Pulse Radiolysis Study of L-Ascorbic Acid-2-Sulfate. *Radiat. Res.* **1983**, *95* (3), 530–540. https://doi.org/10.2307/3576098.

(12) Bielski, B. H. J.; Comstock, D. A.; Bowen, R. A. Ascorbic Acid Free Radicals. I. Pulse Radiolysis Study of Optical Absorption and Kinetic Properties. *J. Am. Chem. Soc.* **1971**, *93* (22), 5624–5629. https://doi.org/10.1021/ja00751a006.

(13) Shen, J.; Griffiths, P. T.; Campbell, S. J.; Utinger, B.; Kalberer, M.; Paulson, S. E. Ascorbate Oxidation by Iron, Copper and Reactive Oxygen Species: Review, Model Development, and Derivation of Key Rate Constants. *Sci. Rep.* **2021**, *11* (1), 1–14. https://doi.org/10.1038/s41598-021-86477-8.

(14) Visilel, J. M.; Schafer, F. Q.; Buettner, G. R. NIH Public Access. *Anal. Biochem.* **2007**, *365* (1), 31–39.

(15) Dewhirst, R. A.; Fry, S. C. The Oxidation of Dehydroascorbic Acid and 2,3-Diketogulonate by Distinct Reactive Oxygen Species. *Biochem. J.* **2018**, *475* (21), 3451–3470. https://doi.org/10.1042/BCJ20180688.

(16) Parsons, H. T.; Yasmin, T.; Fry, S. C. Alternative Pathways of Dehydroascorbic Acid Degradation in Vitro and in Plant Cell Cultures: Novel Insights into Vitamin C Catabolism. *Biochem. J.* **2011**, *440* (3), 375–383. https://doi.org/10.1042/BJ20110939.

(17) De Laat, J.; Gallard, H. Catalytic Decomposition of Hydrogen Peroxide by Fe(III) in Homogeneous Aqueous Solution: Mechanism and Kinetic Modeling. *Environ. Sci. Technol.* **1999**, *33* (16), 2726–2732. https://doi.org/10.1021/es981171v.

(18) He, J.; Yang, X.; Men, B.; Wang, D. Interfacial Mechanisms of Heterogeneous Fenton Reactions Catalyzed by Iron-Based Materials: A Review. *J. Environ. Sci. (China)* **2016**, *39*, 97–109. https://doi.org/10.1016/j.jes.2015.12.003.

(19) Pignatello, J. J.; Oliveros, E.; MacKay, A. Advanced Oxidation Processes for Organic Contaminant Destruction Based on the Fenton Reaction and Related Chemistry. *Crit. Rev. Environ. Sci. Technol.* **2006**, *36* (1), 1–84. https://doi.org/10.1080/10643380500326564.

(20) De Laat, J.; Gallard, H.; Ancelin, S.; Legube, B. Comparative Study of the Oxidation of Atrazine and Acetone by H2O2/UV, Fe(III)/UV, Fe(III)/H2O2/UV and Fe(II) or Fe(III)/H2O2. *Chemosphere* **1999**, *39* (15), 2693–2706. https://doi.org/10.1016/S0045-6535(99)00204-0.

(21) Rokhina, E. V.; Makarova, K.; Lahtinen, M.; Golovina, E. A.; Van As, H.; Virkutyte, J. Ultrasound-Assisted MnO2 Catalyzed Homolysis of Peracetic Acid for Phenol Degradation: The Assessment of Process Chemistry and Kinetics. *Chem. Eng. J.* **2013**, *221*, 476–486. https://doi.org/10.1016/j.cej.2013.02.018.

(22) Rojas, M. R.; Pérez, F.; Whitley, D.; Arnold, R. G.; Sáez, A. E. Modeling of Advanced Oxidation of Trace Organic Contaminants by Hydrogen Peroxide Photolysis and Fentons Reaction. *Ind. Eng. Chem. Res.* **2010**, *49* (22), 11331–11343. https://doi.org/10.1021/ie101592p.

(23) Mártire, D. O.; Caregnato, P.; Furlong, J.; Allegretti, P.; Gonzalez, M. C. Kinetic Study of the Reactions of Oxoiron(IV) with Aromatic Substrates in Aqueous Solutions. *Int. J. Chem. Kinet.* **2002**, *34* (8), 488–494. https://doi.org/10.1002/kin.10076.

(24) Gonzalez, D. H.; Cala, C. K.; Peng, Q.; Paulson, S. E. HULIS Enhancement of Hydroxyl Radical Formation from Fe(II): Kinetics of Fulvic Acid-Fe(II) Complexes in the Presence of Lung Antioxidants. *Environ. Sci. Technol.* **2017**, *51* (13), 7676–7685. https://doi.org/10.1021/acs.est.7b01299.

(25) Miller, C. J.; Rose, A. L.; Waite, T. D. Hydroxyl Radical Production by H2O2-Mediated Oxidation of Fe(II) Complexed by Suwannee River Fulvic Acid under Circumneutral Freshwater Conditions. *Environ. Sci. Technol.* **2013**, *47* (2), 829–835. https://doi.org/10.1021/es303876h.

(26) Pham, A. N.; Waite, T. D. Modeling the Kinetics of Fe(II) Oxidation in the Presence of Citrate and Salicylate in Aqueous Solutions at PH 6.0-8.0 and 25°C. *J. Phys. Chem. A* **2008**, *112* (24), 5395–5405. https://doi.org/10.1021/jp801126p.

(27) De Laat, J.; Le, T. G. Kinetics and Modeling of the Fe(III)/H2O2 System in the Presence of Sulfate in Acidic Aqueous Solutions. *Environ. Sci. Technol.* **2005**, *39* (6), 1811–1818. https://doi.org/10.1021/es0493648.

(28) Herrmann, H.; Tilgner, A.; Barzaghi, P.; Majdik, Z.; Gligorovski, S.; Poulain, L.; Monod, A. Towards a More Detailed Description of Tropospheric Aqueous Phase Organic Chemistry: CAPRAM 3.0. *Atmos. Environ.* **2005**, *39* (23–24), 4351–4363. https://doi.org/10.1016/j.atmosenv.2005.02.016.

(29) Powell, K. J.; Brown, P. L.; Byrne, R. H.; Gajda, T.; Hefter, G.; Sjöberg, S.; Wanner, H. Chemical Speciation of Environmentally Significant Metals with Inorganic Ligands. PART 2: The Cu2+-OH-, Cl-, CO 32-, SO42-, and PO43- Systems (IUPAC Technical Report). *Pure Appl. Chem.* **2007**, *79* (5), 895–950. https://doi.org/10.1351/pac200779050895.

(30) Deguillaume, L., M. Leriche, K. Desboeufs, G. Mailhot, C. G. and N. C. “Transition Metals in Atmospheric Liquid Phases: Sources, Reactivity, and Sensitive Parameters.” *Chem. Rev.* **2005**, *105* (9), 3388–3431.

(31) Lee, H.; Lee, H. J.; Seo, J.; Kim, H. E.; Shin, Y. K.; Kim, J. H.; Lee, C. Activation of Oxygen and Hydrogen Peroxide by Copper(II) Coupled with Hydroxylamine for Oxidation of Organic Contaminants. *Environ. Sci. Technol.* **2016**, *50* (15), 8231–8238. https://doi.org/10.1021/acs.est.6b02067.

(32) Wang, Z.; Liu, Q.; Yang, F.; Huang, Y.; Xue, Y.; Yuan, R.; Sheng, B.; Wang, X. Accelerated Oxidation of 2,4,6-Trichlorophenol in Cu(II)/H2O2/Cl- System: A Unique “Halotolerant” Fenton-like Process? *Environ. Int.* **2019**, *132* (May), 105128. https://doi.org/10.1016/j.envint.2019.105128.

(33) Zhou, P.; Zhang, J.; Zhang, Y.; Liu, Y.; Liang, J.; Liu, B.; Zhang, W. Generation of Hydrogen Peroxide and Hydroxyl Radical Resulting from Oxygen-Dependent Oxidation of l-Ascorbic Acid via Copper Redox-Catalyzed Reactions. *RSC Adv.* **2016**, *6* (45), 38541–38547. https://doi.org/10.1039/c6ra02843h.

(34) Pham, A. N.; Xing, G.; Miller, C. J.; Waite, T. D. Fenton-like Copper Redox Chemistry Revisited: Hydrogen Peroxide and Superoxide Mediation of Copper-Catalyzed Oxidant Production. *J. Catal.* **2013**, *301*, 54–64. https://doi.org/10.1016/j.jcat.2013.01.025.

(35) Charrier, J. G.; McFall, A. S.; Richards-Henderson, N. K.; Anastasio, C. Hydrogen Peroxide Formation in a Surrogate Lung Fluid by Transition Metals and Quinones Present in Particulate Matter. *Environ. Sci. Technol.* **2014**, *48* (12), 7010–7017. https://doi.org/10.1021/es501011w.

(36) Lakey, P. S. J.; Berkemeier, T.; Tong, H.; Arangio, A. M.; Lucas, K.; Pöschl, U.; Shiraiwa, M. Chemical Exposure-Response Relationship between Air Pollutants and Reactive Oxygen Species in the Human Respiratory Tract. *Sci. Rep.* **2016**, *6* (September), 1–6. https://doi.org/10.1038/srep32916.

(37) Roginsky, V. A.; Pisarenko, L. M.; Bors, W.; Michel, C. The Kinetics and Thermodynamics of Quinone-Semiquinone-Hydroquinone Systems under Physiological Conditions. *J. Chem. Soc. Perkin Trans. 2* **1999**, No. 4, 871–876. https://doi.org/10.1039/a807650b.

(38) Roginsky, V. A.; Barsukova, T. K.; Stegmann, H. B. Kinetics of Redox Interaction between Substituted Quinones and Ascorbate under Aerobic Conditions. *Chem. Biol. Interact.* **1999**, *121* (2), 177–197. https://doi.org/10.1016/S0009-2797(99)00099-X.

(39) Yuan, X.; Miller, C. J.; Pham, A. N.; Waite, T. D. Kinetics and Mechanism of Auto- and Copper-Catalyzed Oxidation of 1,4-Naphthohydroquinone. *Free Radic. Biol. Med.* **2014**, *71*, 291–302. https://doi.org/10.1016/j.freeradbiomed.2014.03.021.

(40) Tong, H.; Lakey, P. S. J.; Arangio, A. M.; Socorro, J.; Shen, F.; Lucas, K.; Brune, W. H.; Pöschl, U.; Shiraiwa, M. Reactive Oxygen Species Formed by Secondary Organic Aerosols in Water and Surrogate Lung Fluid. *Environ. Sci. Technol.* **2018**, *52*, 11642–11651. https://doi.org/10.1021/acs.est.8b03695.

(41) Chevallier, E.; Jolibois, R. D.; Meunier, N.; Carlier, P.; Monod, A. "Fenton-like" Reactions of Methylhydroperoxide and Ethylhydroperoxide with Fe2+ in Liquid Aerosols under Tropospheric Conditions. *Atmos. Environ.* **2004**, *38* (6), 921–933. https://doi.org/10.1016/j.atmosenv.2003.10.027.

(42) Fang, T.; Lakey, P. S. J.; Rivera-Rios, J. C.; Keutsch, F. N.; Shiraiwa, M. Aqueous-Phase Decomposition of Isoprene Hydroxy Hydroperoxide and Hydroxyl Radical Formation by Fenton-like Reactions with Iron Ions. *J. Phys. Chem. A* **2020**, *124* (25), 5230–5236. https://doi.org/10.1021/acs.jpca.0c02094.

(43) Gonzalez, D. H.; Cala, C. K.; Peng, Q.; Paulson, S. E. HULIS Enhancement of Hydroxyl Radical Formation from Fe(II): Kinetics of Fulvic Acid-Fe(II) Complexes in the Presence of Lung Antioxidants. *Environ. Sci. Technol.* **2017**, *51* (13), 7676–7685. https://doi.org/10.1021/acs.est.7b01299.

(44) Page, S. E.; Arnold, W. A.; McNeill, K. Terephthalate as a Probe for Photochemically Generated Hydroxyl Radical. *J. Environ. Monit.* **2010**, *12* (9), 1658–1665. https://doi.org/10.1039/c0em00160k.

(45) Goldberg, R. N.; Kishore, N.; Lennen, R. M. Thermodynamic Quantities for the Ionization Reactions of Buffers. *J. Phys. Chem. Ref. Data* **2002**, *31* (2), 231–370. https://doi.org/10.1063/1.1416902.

(46) Skogareva, L., T. Shekunova, A. Baranchikov, A. Yapryntsev, A. Sadovnikov, M. Ryumin, N. M. and V. I. "Synthesis of Cerium Orthophosphates with Monazite and Rhabdophane Structure from Phosphoric Acid Solutions in the Presence of Hydrogen Peroxide." Russian Journal of Inorganic Chemistry 61(10): 1219-1224. *Russ. J. Inorg. Chem.* **2016**, *61* (10), 1219–1224.

(47) Morozov, P Ershov, B. The Influence of Phosphates on the Decomposition of Ozone in Water: Chain Process Inhibition. *Russ. J. Phys. Chem. A* **2010**, *84* (7), 1136–1140.

(48) Kochany, J.; Lipczynska-Kochany, E. Application of the EPR Spin-Trapping Technique for the Investigation of the Reactions of Carbonate, Bicarbonate, and Phosphate Anions with Hydroxyl Radicals Generated by the Photolysis of H2O2. *Chemosphere* **1992**, *25* (12), 1769–1782. https://doi.org/10.1016/0045-6535(92)90018-M.
